# Learning Retention Mechanisms and Evolutionary Parameters of Duplicate Genes from Their Expression Data

**DOI:** 10.1093/molbev/msaa267

**Published:** 2020-10-12

**Authors:** Michael DeGiorgio, Raquel Assis

**Affiliations:** 1 Department of Computer and Electrical Engineering and Computer Science, Florida Atlantic University, Boca Raton, FL 33431; 2 Institute for Human Health and Disease Intervention, Florida Atlantic University, Boca Raton, FL 33431

**Keywords:** gene duplication, neofunctionalization, subfunctionalization, Ornstein–Uhlenbeck, neural network

## Abstract

Learning about the roles that duplicate genes play in the origins of novel phenotypes requires an understanding of how their functions evolve. A previous method for achieving this goal, CDROM, employs gene expression distances as proxies for functional divergence and then classifies the evolutionary mechanisms retaining duplicate genes from comparisons of these distances in a decision tree framework. However, CDROM does not account for stochastic shifts in gene expression or leverage advances in contemporary statistical learning for performing classification, nor is it capable of predicting the parameters driving duplicate gene evolution. Thus, here we develop CLOUD, a multi-layer neural network built on a model of gene expression evolution that can both classify duplicate gene retention mechanisms and predict their underlying evolutionary parameters. We show that not only is the CLOUD classifier substantially more powerful and accurate than CDROM, but that it also yields accurate parameter predictions, enabling a better understanding of the specific forces driving the evolution and long-term retention of duplicate genes. Further, application of the CLOUD classifier and predictor to empirical data from *Drosophila* recapitulates many previous findings about gene duplication in this lineage, showing that new functions often emerge rapidly and asymmetrically in younger duplicate gene copies, and that functional divergence is driven by strong natural selection. Hence, CLOUD represents a major advancement in classifying retention mechanisms and predicting evolutionary parameters of duplicate genes, thereby highlighting the utility of incorporating sophisticated statistical learning techniques to address long-standing questions about evolution after gene duplication.

## Introduction

Gene duplication is a mutational process that creates copies of existing genes. Experimental studies in several diverse species have revealed that duplication occurs faster than all other types of spontaneous mutation (Lynch et al. 2008; Lipinski et al. 2011; Schrider et al. 2013; Keith et al. 2016; Konrad et al. 2018), thus serving as a major reservoir of genetic variation. Moreover, in contrast to other types of mutation, duplication generates redundancy, permitting the exploration of evolutionary space that may have been ancestrally forbidden (Ohno 1970). As a result, duplication has long been hypothesized to underlie the origins of novel phenotypes and complex biological systems (Ohno 1970). Indeed, mounting evidence of widespread duplication and its contribution to adaptation and speciation in all three biological domains (Zhang 2003; Kondrashov 2012) highlights its key role in evolution across the tree of life.

**Fig. 1:**
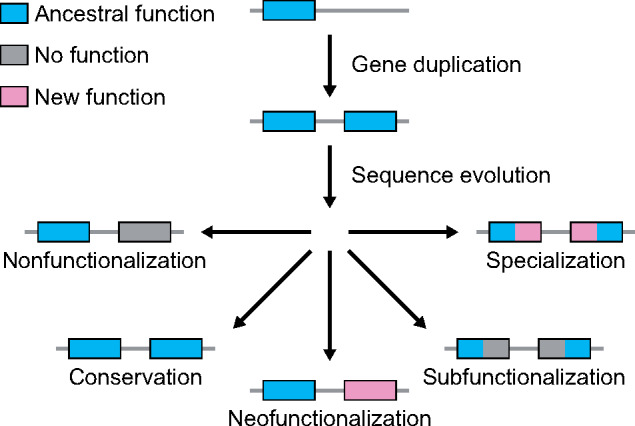
Hypothesized evolutionary trajectories of duplicate genes. Gene duplication results in two copies of an ancestral gene. Evolution may result in the loss of one functional copy by nonfunctionalization, or in the retention of two functional copies by either conservation, neofunctionalization, subfunctionalization, or specialization.

Yet, the evolutionary path leading from gene duplication to functional innovation remains unclear. According to traditional evolutionary models (Ohno 1970; Force et al. 1999; Stoltzfus 1999; Lynch and Force 2000; He and Zhang 2005; Rastogi and Liberles 2005), gene duplication generates a younger “child” copy that is identical to its older “parent” copy (fig. 1). Though such redundancy can promote adaptation through a relaxation of selective constraint (Ohno 1970), beneficial mutations are rare (Lynch and Force 2000). Hence, theory predicts that the most common outcome of gene duplication is nonfunctionalization, whereby one copy loses its function via an accumulation of deleterious mutations, leading to a reversion back to the ancestral single-copy state (Lynch and Force 2000). As a result, four mechanisms have been proposed to explain how numerous duplicate genes bypass nonfunctionalization and are retained over millions of years of evolution (Ohno 1970; Zhang 2003; Force et al. 1999; Stoltzfus 1999; He and Zhang 2005; Rastogi and Liberles 2005). First, either benefits of increased gene dosage (Ohno 1970) or recombination between gene copies (Zhang 2003) may result in conservation, whereby both copies maintain the ancestral function. Second, beneficial mutations in one gene copy may lead to neofunctionalization, whereby this copy acquires a new function while the other maintains the ancestral function (Ohno 1970). Third, deleterious mutations targeting different functional domains of each gene copy may result in subfunctionalization, whereby each copy maintains a distinct subset of the ancestral function (Force et al. 1999; Stoltzfus 1999). Fourth, a combination of deleterious and beneficial mutations targeting different functional domains of each gene copy may lead to specialization, whereby each copy maintains a subset of the ancestral function and also acquires a new function (He and Zhang 2005; Rastogi and Liberles 2005). Though mutations initiating the latter three retention mechanisms may take some time to appear, dosage balance can act as an intermediate state for preventing gene loss through nonfunctionalization during this waiting period (Hughes et al. 2007; Veitia et al. 2008; Edger and Pires 2009; Teufel et al. 2016; Raju 2020).

On the other hand, genomic studies from the past two decades show that the duplication process itself can often generate a child copy that is distinct from its parent copy (Cusack and Wolfe 2006; Hakes et al. 2007; Assis and Bachtrog 2013; Assis 2014; Cardoso-Moreira et al. 2016; Rogers et al. 2017). A key example is RNA-mediated duplication, which creates a child copy with its parent’s protein-coding sequence, but missing its introns and regulatory elements (Cusack and Wolfe 2006; Assis and Bachtrog 2013; Assis 2014). Duplicate gene copies arising from RNA-mediated duplication frequently display immediate sequence and expression differentiation consistent with functional independence (Cusack and Wolfe 2006; Assis and Bachtrog 2013; Assis 2014). Such differences are also more common after small-scale than whole-genome duplication events (Hakes et al. 2007), perhaps due to incomplete copying of short- and long-range regulatory elements, as well as after complex duplication events involving shuffling of exons and regulatory elements (Rogers et al. 2017). Thus, asymmetric duplication may directly contribute to the emergence of novel gene functions by reducing or removing the waiting period for new mutations required under traditional neofunctionalization, subfunctionalization, and specialization models (Ohno 1970; Zhang 2003; Force et al. 1999; Stoltzfus 1999; He and Zhang 2005; Rastogi and Liberles 2005) (fig. 1).

Due to their vastly different evolutionary forces and functional outcomes, differentiating among duplicate gene retention mechanisms is critical to understanding how gene duplication drives phenotypic innovation. Accordingly, many studies have tackled this problem through applications of comparative (Kondrashov et al. 2002; Kellis et al. 2004; He and Zhang 2005) and model-based approaches (Hughes and Liberles 2007; Konrad et al. 2011) to DNA sequence data. However, perhaps a more direct source of functional information about a gene is its expression profile, which captures its activity levels across multiple conditions (e.g., tissues, developmental stages, or disease states). In particular, gene expression profiles are ideal proxies for function due to their correlations with many other functional metrics, including protein-coding gene sequence divergence (Nuzhdin et al. 2004; Subramanian and Kumar 2004; Lemos et al. 2005; Hunt et al. 2013; Assis and Kondrashov 2014; Mahler et al. 2017; Assis 2019a), protein–protein interaction networks (Ge et al. 2001; Bhardwaj and Lu 2005; Lemos et al. 2005; French and Pavlidis 2011; Assis and Kondrashov 2014; Mahler et al. 2017; Assis 2019a), and biological processes and pathways (Zhou et al. 2002; Assis 2019a). Moreover, high-throughput gene expression data are widely available for numerous conditions and species, and simple to quantify and compare relative to alternative proxies of gene function.

With this in mind, Assis and Bachtrog (2013) designed a decision tree classification algorithm based on comparisons of differences between multi-tissue expression profiles of ancestral single-copy genes and their derived parent and child duplicate gene copies. Their approach (Assis and Bachtrog 2013), which was later generalized to other types of input data and implemented in the R package CDROM (Perry and Assis 2016), has been used to classify retention mechanisms of duplicate genes in *Drosophila* (Assis and Bachtrog 2013), mammals (Assis and Bachtrog 2015), honeybees (Chau and Goodisman 2017), and grasses (Jiang and Assis 2019). Together, these studies have demonstrated that duplicate genes are frequently retained by neofunctionalization (Assis and Bachtrog 2013, 2015; Assis 2014; Chau and Goodisman 2017; Jiang and Assis 2019), that child copies more often acquire new functions than parent copies (Assis and Bachtrog 2013, 2015; Assis 2014; Jiang and Assis 2019), and that new functions tend to be male-specific (Assis and Bachtrog 2013, 2015; Assis 2014; Chau and Goodisman 2017; Jiang and Assis 2019). These findings are concordant with earlier work showing that young animal and plant duplicate genes are often specifically expressed in male tissues (Betrán et al. 2002; Marques et al. 2005; Kaessmann 2010; Zhang et al. 2010; Wu et al. 2014). Further, earlier studies showed that levels of protein-coding sequence divergence are often consistent with retention mechanisms classified based on gene expression divergence (Assis and Bachtrog 2013, 2015; Assis 2014; Chau and Goodisman 2017), and a follow-up analysis in *Drosophila* revealed natural selection to play important roles in both whether and how duplicate genes are retained over evolutionary time (Jiang and Assis 2017).

However, there are two major shortcomings of the method implemented by CDROM (Assis and Bachtrog 2013; Perry and Assis 2016). First, it does not account for stochastic shifts in gene expression that may occur as a result of phenotypic drift (Oleksiak et al. 2002; Khaitovich et al. 2004). Second, it does not leverage the power provided by recent advances in statistical and machine learning (Hastie et al. 2009; Goodfellow et al. 2016). With these limitations in mind, we developed CLassification using Ornstein–Uhlenbeck of Duplicates (CLOUD), a novel classification algorithm that employs simulated training data generated by Ornstein–Uhlenbeck (OU) processes, which can model gene expression evolution along phylogenetic trees (Hansen 1997; Butler and King 2004; Bedford and Hartl 2008; Kalinka et al. 2010; Brawand et al. 2011; Perry et al. 2012; Rohlfs et al. 2014; Rohlfs and Nielsen 2015). In particular, because OU processes model Brownian motion with a pull toward an optimal state, they have a natural application to evolution, in which phenotypic drift is analogous to Brownian motion, natural selection to pull, and fittest phenotype to optimal state (Hansen 1997; Butler and King 2004).

Though OU processes have been used to model expression evolution of single-copy genes (Bedford and Hartl 2008; Kalinka et al. 2010; Brawand et al. 2011; Perry et al. 2012; Rohlfs et al. 2014; Rohlfs and Nielsen 2015), they have never been applied to the analogous problem after gene duplication. Thus, CLOUD adapts the OU framework to quantify expression evolution after gene duplication by modeling changes along a tree relating a pair of duplicate genes (parent and child copies) and their ancestral gene in a related sister species. Then, it utilizes the simulated output of these models to construct a multilayer feed-forward neural network for classifying duplicate genes as retained under conservation, neofunctionalization, subfunctionalization, or specialization. Moreover, this approach enables CLOUD to also predict parameters influencing the expression evolution of duplicate genes. Application of CLOUD to simulated data shows that it has high power to differentiate among classes, vastly outperforming CDROM for a wide range of parameter values, and also accurately predicts parameters shaping the expression evolution of retained duplicate genes. Further, application of CLOUD to empirical data from *Drosophila* (Assis and Bachtrog 2013; Assis 2019b) recapitulates a majority of the classified duplicate gene retention mechanisms presented by Assis and Bachtrog (2013), as well as generates parameter predictions that match theoretical expectations of these retention mechanisms. CLOUD has been implemented as an R package, and is freely available at http://assisgroup.fau.edu/software.html and https://github.com/rassis/CLOUD. Its input data can include gene expression measured for a single condition or multiple conditions of varying types (e.g., tissues, developmental stages, or disease states), making it applicable to a wide range of single- and multi-cellular biological systems.

## Results

In this section, we design our CLOUD classifier and predictor, evaluate its performance on simulated data, and apply it to an empirical data set from *Drosophila*. First, we introduce our OU framework for modeling expression evolution after gene duplication, which forms the basis of the CLOUD classifier and predictor. Next, we formally define the multilayer neural network architecture implemented by CLOUD for both classification and prediction tasks. We then employ simulations to evaluate the relative classification powers and accuracies of CDROM and CLOUD across a wide range of parameters, as well as in more targeted regions of the parameter space. We also use these simulations to probe its accuracy in predicting parameters driving gene expression evolution after duplication, specifically its ability to estimate optimal gene expression, selection strength, and phenotypic drift for each of the classified retention mechanisms. Last, we apply CLOUD to empirical data from *Drosophila* (Assis and Bachtrog 2013; Assis 2019b) to classify retention mechanisms and predict underlying evolutionary parameters after gene duplication in this lineage.

### Modeling Expression Evolution after Gene Duplication as an OU Process

**Fig. 2:**
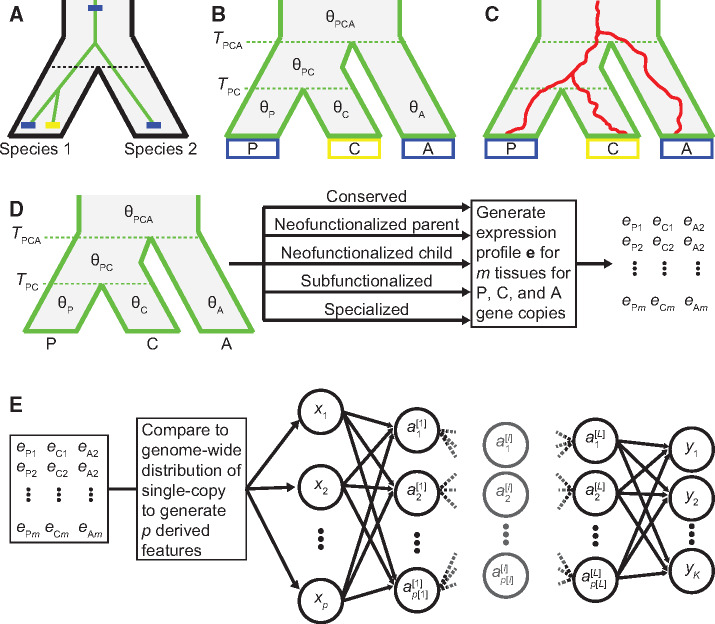
Modeling expression evolution after gene duplication as an OU process. (*A*) Relationships between two species (black phylogeny) and their genes (green phylogeny). After the two species diverged, a blue gene in Species 1 (parent) underwent a duplication event to create a yellow copy (child). (*B*) Relationships among the parent gene copy (P) in Species 1, child gene copy (C) in Species 1, and ancestral single-copy gene (A) in Species 2. The duplication event occurred at time $T_{\text{PC}}$, and both copies split from the ancestral gene at time $T_{\text{PCA}}$. Optimal expression states for the parent, child, and ancestral genes are given by $\theta_{P},\;\theta_{C}$, and $\theta_{A}$, respectively. The internal branch and the branch above the root have optimal expression states $\theta_{\text{PC}}$ and $\theta_{\text{PCA}}$, respectively. (*C*) Cartoon depicting expression profile changes (red lines) along the gene tree. Expression profiles change randomly through phenotypic drift with strength $\sigma^{2}$, and toward the optimal expression state through selection with strength *α*. (*D*) Illustration of how we simulate multi-tissue expression vectors for parent, child, and ancestral genes. (*E*) Schematic of our feed-forward neural network architecture, which takes in *p* input units with values $x_{1},x_{2},\ldots ,x_{p}$, has *K* output units with values $y_{1},y_{2},\ldots ,y_{K}$, and has *L* hidden layers, where the number of units in layer ℓ is p[ℓ] and the value of unit *k* in layer ℓ is the activation $a_{k}^{\left[\ell \right]}$.

To design a model of expression evolution after gene duplication, we consider a pair of related species, Species 1 and Species 2, whose lineages diverged from that of a common ancestor at time $T_{\text{PCA}}$ (fig. 2*A*–*C*). Suppose that the common ancestor had a single-copy gene that underwent duplication, giving rise to a pair of duplicate genes at time $T_{\text{PC}}$ in the lineage of Species 1 after its divergence from the lineage of Species 2. Of the pair of duplicate genes in Species 1, we designate the copy corresponding to the original single-copy gene in the ancestor and in Species 2 as the parent, and the new copy that is absent in both the ancestor and Species 2 as the child. Further, suppose that optimal expression states for the parent, child, and ancestral genes are given by $\theta_{P},\;\theta_{C}$, and $\theta_{A}$, respectively. Likewise, the optimal expression state for the single-copy gene in the ancestor prior to the divergence of Species 1 and Species 2 is given as $\theta_{\text{PCA}}$, and for the single-copy gene in the lineage of Species 1 before duplication occurred as $\theta_{\text{PC}}$. Additionally, assume that $\theta_{\text{PC}}=\theta_{\text{PCA}}=\theta_{A}$. We then model expression along the tree relating the parent, child, and ancestral genes as changing randomly through phenotypic drift with strength $\sigma^{2}$, and toward the optimal expression state through selection with strength *α*, according to an OU process.

In each tissue, gene expression $e=(e_{P},e_{C},e_{A})\in {\mathbb{R}}^{3}$ is therefore distributed as a multivariate normal (MVN) distribution with mean

$$\mu =\left(E[e_{P}],E[e_{C}],E[e_{A}]\right)\in {\mathbb{R}}^{3}$$

and covariance matrix

$$\Sigma =\left[\begin{array}{ccc} \mathrm{Var}[e_{P}] & \mathrm{Cov}[e_{P},e_{C}] & \mathrm{Cov}[e_{P},e_{A}]\\ \mathrm{Cov}[e_{C},e_{P}] & \mathrm{Var}[e_{C}] & \mathrm{Cov}[e_{C},e_{A}]\\ \mathrm{Cov}[e_{A},e_{P}] & \mathrm{Cov}[e_{A},e_{C}] & \mathrm{Var}[e_{C}] \end{array}\right]\in {\mathbb{R}}^{3\times 3},$$

that is, e∼MVN(μ,Σ). Following Brawand et al. (2011), we have that

$$\mu =\left[\begin{array}{c} \left(1-e^{-\alpha T_{\text{PC}}}\right)\theta_{P}+e^{-\alpha T_{\text{PC}}}\theta_{A}\\ \left(1-e^{-\alpha T_{\text{PC}}}\right)\theta_{C}+e^{-\alpha T_{\text{PC}}}\theta_{A}\\ \theta_{A} \end{array}\right]$$

and

$$\Sigma =\frac{\sigma^{2}}{2\alpha }\left[\begin{array}{ccc} 1 & e^{-2\alpha T_{\text{PC}}} & e^{-2\alpha T_{\text{PCA}}}\\ e^{-2\alpha T_{\text{PC}}} & 1 & e^{-2\alpha T_{\text{PCA}}}\\ e^{-2\alpha T_{\text{PCA}}} & e^{-2\alpha T_{\text{PCA}}} & 1 \end{array}\right].$$

Here, we assume that expression is independent across tissues. However, this approach can also be extended to account for the intertissue expression covariance structure using established approaches (Revell and Harmon 2008; Revell and Collar 2009; Clavel et al. 2015).

### Neural Network Architecture for the CLOUD Classifier and Predictor

We denote the set of all genes with two copies in one species and one copy in the other as duplicate genes D, and the set of all genes with one copy in both species as single-copy genes G. Let

$$e^{\left(d\right)}=\left(e_{P1}^{\left(d\right)},e_{C1}^{\left(d\right)},e_{A1}^{\left(d\right)},\ldots ,e_{Pm}^{\left(d\right)},e_{Cm}^{\left(d\right)},e_{Am}^{\left(d\right)}\right)\in {\mathbb{R}}^{3m}$$

be the input expression vector for duplicate gene d∈D across *m* tissues, where $e_{jk}^{\left(d\right)}$ is the expression level for copy j∈{P,C,A} of duplicate gene *d* in tissue k∈{1,2,…,m}. Similarly, let

$$s^{\left(g\right)}=\left(s_{11}^{\left(g\right)},s_{21}^{\left(g\right)},\ldots ,s_{1m}^{\left(g\right)},s_{2m}^{\left(g\right)}\right)\in {\mathbb{R}}^{2m}$$

be the expression vector for single-copy gene g∈G across *m* tissues, where $s_{jk}^{\left(g\right)}$ is the expression level for species j∈{1,2} of single-copy gene *g* in tissue *k*.

We transform and compare the expression vector $e^{\left(d\right)}$ of each duplicate gene d∈D to the expression vector $s^{\left(g\right)}$ of each single-copy gene g∈G to obtain the feature vector

$$x^{\left(d\right)}=\left(x_{1}^{\left(d\right)},x_{2}^{\left(d\right)},\ldots ,x_{p}^{\left(d\right)}\right)\in {\mathbb{R}}^{p},$$

which we use as input to a dense feed-forward neural network. Following Assis and Bachtrog (2013), we compare multi-tissue expression differences between duplicate genes D to the distribution of multi-tissue expression differences between single-copy genes G. Specifically, we generate the set of p=4m+84 derived features listed in table 1, many of which involve comparisons to distributions of values for single-copy genes G. To generate these distributions, we compute the Euclidean distance and Pearson correlation coefficient between the multi-tissue expression vectors of Species 1 and Species 2 for each single-copy gene g∈G. Based on these values, we derive the sets of all Euclidean distances dist(G) and Pearson correlation coefficients cor(G). We utilize features based on both dist(G) and cor(G) so that we can evaluate not only differences among values, but also among their shapes (Hastie et al. 2009).

**Table 1:** Set of p=4m+84 derived features used as input to CLOUD.

Feature number	Feature value
1	$t_{\text{PC}}=T_{\text{PC}}/T_{\text{PCA}}$
2 to 3m+1	$e^{\left(d\right)}=\left(e_{P1}^{\left(d\right)},e_{C1}^{\left(d\right)},e_{A1}^{\left(d\right)},\ldots ,e_{Pm}^{\left(d\right)},e_{Cm}^{\left(d\right)},e_{Am}^{\left(d\right)}\right)$
3 *m *+* *2 to 4m+1	$e_{P1}^{\left(d\right)}+e_{C1}^{\left(d\right)},\ldots ,e_{Pm}^{\left(d\right)}+e_{Cm}^{\left(d\right)}$
4 *m *+* *2	$\text{dist}(P,C)=\sqrt{{\left(e_{P1}^{\left(d\right)}-e_{C1}^{\left(d\right)}\right)}^{2}+\cdots +{\left(e_{Pm}^{\left(d\right)}-e_{Cm}^{\left(d\right)}\right)}^{2}}$
4 *m *+* *3	$\text{dist}(P,A)=\sqrt{{\left(e_{P1}^{\left(d\right)}-e_{A1}^{\left(d\right)}\right)}^{2}+\cdots +{\left(e_{Pm}^{\left(d\right)}-e_{Am}^{\left(d\right)}\right)}^{2}}$
4 *m *+* *4	$\text{dist}(C,A)=\sqrt{{\left(e_{C1}^{\left(d\right)}-e_{A1}^{\left(d\right)}\right)}^{2}+\cdots +{\left(e_{Cm}^{\left(d\right)}-e_{Am}^{\left(d\right)}\right)}^{2}}$
4 *m *+* *5	$\text{dist}(\text{PC},A)=\sqrt{{\left(e_{P1}^{\left(d\right)}+e_{C1}^{\left(d\right)}-e_{A1}^{\left(d\right)}\right)}^{2}+\cdots +{\left(e_{Pm}^{\left(d\right)}+e_{Cm}^{\left(d\right)}-e_{Am}^{\left(d\right)}\right)}^{2}}$
4 *m *+* *6	branch(P)=[dist(P,C)+dist(P,A)−dist(C,A)]/2
4 *m *+* *7	branch(C)=[dist(P,C)+dist(C,A)−dist(P,A)]/2
4 *m *+* *8	branch(A)=[dist(P,A)+dist(C,A)−dist(P,C)]/2
4 *m *+* *9	rank of dist(P,C) among dist(G)
4 *m *+* *10	rank of dist(P,A) among dist(G)
4 *m *+* *11	rank of dist(C,A) among dist(G)
4 *m *+* *12	rank of dist(PC,A) among dist(G
4 *m *+* *13 to 4 *m *+* *20	*k*th moment of [dist(P,C)−dist(G)]/max⁡{dist(G)} for k=1,2,…,8
4*m* + 21 to 4 *m *+* *28	*k*th moment of [dist(P,A)−dist(G)]/max⁡{dist(G)} for k=1,2,…,8
4*m* + 29 to 4 *m *+* *36	*k*th moment of [dist(C,A)−dist(G)]/max⁡{dist(G)} for k=1,2,…,8
4*m* + 37 to 4 *m *+* *44	*k*th moment of [dist(PC,A)−dist(G)]/max⁡{dist(G)} for k=1,2,…,8
4*m* + 45	$\text{cor}(P,C)=\text{PearsonCorrelation}\left(e_{P1}^{\left(d\right)},\ldots ,e_{Pm}^{\left(d\right)};e_{C1}^{\left(d\right)},\ldots ,e_{Cm}^{\left(d\right)}\right)$
4 *m *+* *46	$\text{cor}(P,A)=\text{PearsonCorrelation}\left(e_{P1}^{\left(d\right)},\ldots ,e_{Pm}^{\left(d\right)};e_{A1}^{\left(d\right)},\ldots ,e_{Am}^{\left(d\right)}\right)$
4 *m *+* *47	$\text{cor}(C,A)=\text{PearsonCorrelation}\left(e_{C1}^{\left(d\right)},\ldots ,e_{Cm}^{\left(d\right)};e_{A1}^{\left(d\right)},\ldots ,e_{Am}^{\left(d\right)}\right)$
4 *m *+* *48	$\text{cor}(\text{PC},A)=\text{PearsonCorrelation}\left(e_{P1}^{\left(d\right)}+e_{C1}^{\left(d\right)},\ldots ,e_{Pm}^{\left(d\right)}+e_{Cm}^{\left(d\right)};e_{A1}^{\left(d\right)},\ldots ,e_{Am}^{\left(d\right)}\right)$
4 *m *+* *49	rank of cor(P,C) among cor(G)
4 *m *+* *50	rank of cor(P,A) among cor(G)
4 *m *+* *51	rank of cor(C,A) among cor(G)
4 *m *+* *52	rank of cor(PC,A) among cor(G)
4 *m *+* *53 to 4 *m *+* *60	*k*th moment of cor(P,C)−cor(G) for k=1,2,…,8
4*m* + 61 to 4 *m *+* *68	*k*th moment of cor(P,A)−cor(G) for k=1,2,…,8
4*m* + 69 to 4 *m *+* *76	*k*th moment of cor(C,A)−cor(G) for k=1,2,…,8
4*m* + 77 to 4 *m *+* *84	*k*th moment of cor(PC,A)−cor(G) for k=1,2,…,8

Given the input feature vector $x^{\left(d\right)}$, we seek to predict the output vector

$$y^{\left(d\right)}=\left(y_{1}^{\left(d\right)},y_{2}^{\left(d\right)},\ldots ,y_{K}^{\left(d\right)}\right)\in {\mathbb{R}}^{K}.$$

When performing classification of duplicate gene retention mechanisms, $y^{\left(d\right)}$ is the vector of *K *=* *5 class probabilities, corresponding to class labels “Conserved” for conservation, “Neofunctionalized parent” for neofunctionalization in which the parent copy acquires a new function, “Neofunctionalized child” for neofunctionalization in which the child copy acquires a new function, “Subfunctionalized” for subfunctionalization, and “Specialized” for specialization. In contrast, when predicting evolutionary parameters of duplicate genes, $y^{\left(d\right)}$ is the vector of K=5m parameter predictions in each of the *m* tissues, where in each tissue we obtain parameter estimates for $\theta_{P},\;\theta_{C},\;\theta_{A},\;\sigma^{2}$, and *α*.

We consider a dense feed-forward neural network with L∈{0,1,2,3} hidden layers. The first hidden layer has p[1]=256 hidden units, and hidden layer ℓ∈{1,2,…,L} has $p[\ell ]=256/2^{\ell -1}$ hidden units, such that each hidden layer has half the number of hidden units as the previous hidden layer. For the purposes of condensing notation, we also consider the input layer as hidden layer zero, such that p[0]=p=4m+84 is the number of input features, and we consider the output layer as hidden layer *L *+* *1, such that p[L+1]=K.

We define the values at unit k∈{1,2,…,p[ℓ]} of hidden layer ℓ∈{0,1,2,…,L} for duplicate gene d∈D by its activation $a_{k}^{\left(d)[\ell \right]}$. Because hidden layer zero is the input layer and hidden layer *L *+* *1 is the output layer, then

$$\begin{array}{c} a_{k}^{\left(d)[0\right]}=x_{k}^{\left(d\right)}\\ y_{k}^{\left(d\right)}=a_{k}^{\left(d)[L+1\right]}. \end{array}$$

For hidden layer ℓ∈{1,2,…,L}, we define the activation for unit *k* as a linear combination of the activations from the previous hidden layers, followed by a non-linear transformation (Goodfellow et al. 2016). Here we choose the rectified linear unit (ReLU; Goodfellow et al. 2016) function defined as ReLU(x)=max⁡(0,x), such that the activation for unit *k* in hidden layer ℓ of duplicate gene *d* is

$$a_{k}^{\left(d)[\ell \right]}=\text{ReLU}\left(w_{0}^{\left[\ell -1\right]}+\sum_{j=1}^{p[\ell -1]}w_{jk}^{\left[\ell -1\right]}a_{j}^{\left(d)[\ell -1\right]}\right),$$

where $w_{jk}^{\left[\ell \right]}\in \mathbb{R}$ is the weight (parameter) from unit *j* in layer ℓ to unit *k* in layer ℓ+1, and where $w_{0}^{\left[\ell \right]}$ is the bias for layer ℓ (Goodfellow et al. 2016). The output layer takes inputs from layer *L*, and has a different form depending on whether we consider the classification or the prediction problem. For the classification problem, we employ the softmax activation function (Goodfellow et al. 2016), such that the output for class k∈{1,2,…,K} of duplicate gene *d* is the probability

$$y_{k}^{\left(d\right)}=\frac{\;\text{exp}\;\left(w_{0}^{\left[L\right]}+\sum_{j=1}^{p[L]}w_{jk}^{\left[L\right]}a_{j}^{\left(d)[L\right]}\right)}{\sum_{t=1}^{K}\;\text{exp}\;\left(w_{0}^{\left[L\right]}+\sum_{j=1}^{p[L]}w_{jt}^{\left[L\right]}a_{j}^{\left(d)[L\right]}\right)}.$$

For the prediction problem, we instead use the linear activation function (Goodfellow et al. 2016), such that the output for parameter prediction k∈{1,2,…,5m} of duplicate gene *d* is

$$y_{k}^{\left(d\right)}=w_{0}^{\left[L\right]}+\sum_{j=1}^{p[L]}w_{jk}^{\left[L\right]}a_{j}^{\left(d)[L\right]}.$$

This neural network was implemented in R (R Core Team 2013), using Keras (Allaire and Chollet 2017) with a TensorFlow backend (Abadi et al. 2015). A schematic of the neural network architecture is provided in figure 2*E*. Note that when *L *=* *0, the neural network simplifies to a multinomial regression model (Hastie et al. 2009) for the classification problem, and to a linear regression model (Hastie et al. 2009) for the prediction problem.

### Classification Power and Accuracy of CLOUD Relative to CDROM

To evaluate the classification power and accuracy of our multi-layer neural network classifier CLOUD, we trained and tested it on independent data sets simulated under each class of duplicate gene retention mechanisms (see Materials and Methods section). We assumed two hidden layers when training and testing CLOUD, as this resulted in the best cross-validation performance (see Materials and Methods section). Our training set consisted of 50,000 observations, of which 10,000 were simulated under each class. We trained CLOUD on these data, and explored evolutionary parameters drawn on a logarithmic scale across many orders of magnitude. Specifically, we independently drew the five parameters $\theta_{P},\theta_{C},\theta_{A}\in [10^{-4},10^{4}],\;\alpha \in [1,10^{3}]$, and $\sigma^{2}\in [10^{-2},10^{3}]$ for each of the six tissues, for a total of 30 random parameters per simulated replicate. We then tested CLOUD on a separate set of 5,000 observations, of which 1,000 were simulated under each class, with evolutionary parameters drawn from the same broad space as that of the training set. For comparison, we also applied the existing classifier CDROM (Assis and Bachtrog 2013; Perry and Assis 2016) to the same simulated test set (see Materials and Methods section).

**Fig. 3:**
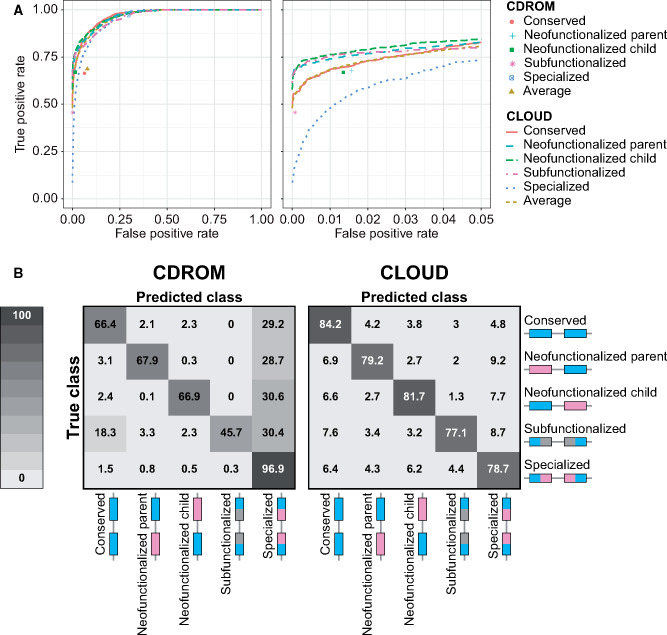
Classification results for CDROM and CLOUD with *L *=* *2 hidden layers applied to data simulated under parameters $\alpha \in [1,10^{3}]$ and $\sigma^{2}\in [10^{-2},10^{3}]$. (*A*) Receiver operating characteristic curves across the full range of false positive rates (left) and truncated at a false positive rate of 5% (right). Because CDROM is a decision tree classifier, its true positive and false positive rates are plotted as points. (*B*) Confusion matrices depicting the classification rates of each of the five duplicate gene retention classes for CDROM (left) and CLOUD (right).

Analysis of the resulting classifications reveals that CLOUD generally has substantially higher power (fig. 3*A*) and accuracy (fig. 3*B*) than CDROM. Specifically, across the wide range of test parameter values explored, CLOUD achieved an accuracy of 80.18%, whereas CDROM only reached 68.76% accuracy (fig. 3*B*). This represents a boost in over 16% classification accuracy of CLOUD relative to CDROM. In addition to increased overall accuracy, CLOUD yields similar accuracies across classes, illustrated by narrow ranges of correct classification rates (between 77.1 and 84.2%; diagonal cells of fig. 3*B*) and mis-classification rates (between 1.3 and 9.2%; non-diagonal cells of fig. 3*B*). In contrast, CDROM demonstrates a much higher correct classification rate for the “Specialized” class (96.9%) than for other classes (between 45.7 and 67.9%; fig. 3*B*), and a higher mis-classification rate toward the “Specialized” class (between 28.7 and 30.6%; fig. 3*B*). Moreover, CDROM experiences additional issues when classifying true “Subfunctionalized” observations, with an 18.3% mis-classification rate toward the “Conserved” class (fig. 3*B*).

In addition, CLOUD is much more conservative than CDROM for pairs of *α* and $\sigma^{2}$ values that are difficult to classify (supplementary figs. S1–S4, Supplementary Material online). For example, both methods typically have higher power (supplementary figs. S3 and S4, Supplementary Material online) and accuracy (supplementary figs. S1 and S2, Supplementary Material online) when either selection is strong (large *α*) or random phenotypic drift is weak (small $\sigma^{2}$). In contrast, when selection is weak (small *α*) and phenotypic drift is strong (large $\sigma^{2}$), then classification is more difficult for both methods. However, in these cases, CLOUD tends to choose classes at similar rates, whereas CDROM is overconfident and chooses the “Specialized” class regardless of the true class (compare supplementary figs. S1 and S2, Supplementary Material online). Therefore, CLOUD not only demonstrates uniformly higher power and accuracy than CDROM across a wide array of evolutionary settings but is also unbiased unlike CDROM.

### Parameter Prediction Accuracy of CLOUD

In addition to its vastly improved classification performance relative to CDROM, a unique attribute of CLOUD is its ability to learn parameters underlying the expression evolution of duplicate genes. Thus, we next assessed the accuracy of the CLOUD predictor by training and testing it on the same independent simulated data sets that we employed for training and testing the CLOUD classifier. In particular, we trained CLOUD (again assuming two hidden layers) to make predictions for each of the five parameters ($\theta_{P},\;\theta_{C},\;\theta_{A}$, *α*, and $\sigma^{2}$) in six tissues (total of 30 parameters) from the training set, and then applied it to make predictions for these parameters from the test set (see Materials and Methods section).

**Fig. 4:**
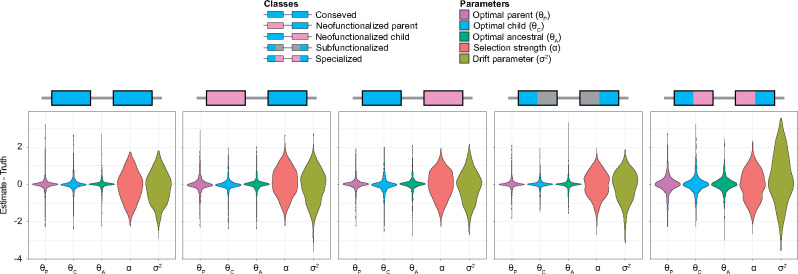
Prediction results for application of CLOUD with *L *=* *2 hidden layers to data simulated under parameters $\alpha \in [1,10^{3}]$ and $\sigma^{2}\in [10^{-2},10^{3}]$ for each of the five classes of duplicate gene retention mechanisms. Violin plots display distributions of mean parameter prediction errors across the *m *=* *6 tissues for each simulated test set.

To investigate prediction accuracy, we examined the distributions of mean parameter prediction errors across the six tissues (fig. 4). In general, all parameter estimates appear unbiased, with mean prediction errors centered on zero. Moreover, estimates of optimal expression states ($\theta_{P},\;\theta_{C}$, and $\theta_{A}$) are more precise than those of selection strength (*α*), which are more precise than those of phenotypic drift ($\sigma^{2}$). Further, parameter predictions for the “Specialized” class are less precise than those for other classes, likely due to the additional degrees of freedom in estimating parameters for this class. In particular, for the “Specialized” class, all optimal expression values are unconstrained (table 2), whereas at least two of the three optimal expression states are constrained to be identical in the “Conserved” and “Neofunctionalized” classes, and $\theta_{P}$ and $\theta_{C}$ are constrained to sum to $\theta_{A}$ for the “Subfunctionalized” class (table 2).

**Table 2:** Optimal expression states under OU processes used to simulate the five classes of duplicate gene retention mechanisms.

Class	Optimal expression state at a given tissue
Conserved	$\theta_{P}=\theta_{C}=\theta_{A}=\theta$
Neofunctionalized parent	$\theta_{C}=\theta_{A}=\theta$ and $\theta_{P}\neq \theta$
Neofunctionalized child	$\theta_{P}=\theta_{A}=\theta$ and $\theta_{C}\neq \theta$
Subfunctionalized	$\theta_{A}=\theta ,\;\theta_{P}\neq \theta ,\;\theta_{C}\neq \theta$, and $\theta_{P}+\theta_{C}=\theta$
Specialized	$\theta_{A}=\theta ,\;\theta_{P}\neq \theta ,\;\theta_{C}\neq \theta$, and $\theta_{P}+\theta_{C}\neq \theta$

As with classification, confidence in parameter predictions made by CLOUD also vary with *α* and $\sigma^{2}$ (supplementary fig. S5, Supplementary Material online). Though precision in estimation tends to be highest when selection is strong (large *α*) or phenotypic drift is weak (small $\sigma^{2}$), it decreases as selection becomes weaker (smaller *α*) or phenotypic drift becomes stronger (larger $\sigma^{2}$). Further, as with our general results across a wide parameter space (fig. 4), estimates of optimal expression states ($\theta_{P},\;\theta_{C}$, and $\theta_{A}$) appear to be unbiased even in narrow regions of the space, with mean prediction errors centered on zero. In contrast, estimates of *α* and $\sigma^{2}$ are biased for some pairs of values. Specifically, estimates of *α* and $\sigma^{2}$ are both upwardly biased for weak selection (small *α*) with weak phenotypic drift (small $\sigma^{2}$), and downwardly biased for strong selection (large *α*) with strong phenotypic drift (large $\sigma^{2}$).

### CLOUD Behavior under Non-uniform Retention Mechanisms across Tissues

We showed that under ideal settings, CLOUD is a superior classifier to CDROM, and is also adept at predicting underlying evolutionary parameters. Thus, we next explored the performance of the trained CLOUD classifier and predictor on test data generated under scenarios that violated model assumptions of the training data. In particular, we considered test data in which the duplicate gene retention mechanism was non-uniform across the simulated tissues. Specifically, we evaluated scenarios in which k∈{1,2,…,m−1} tissues shared one retention mechanism (denoted Tissue Mechanism A) and the remaining *m* − *k* tissues shared a different mechanism (denoted Tissue Mechanism B). As for the trained CLOUD classifier and predictor, we assumed *m *=* *6 tissues, and explored all possible distinct scenarios in which *k* tissues shared one mechanism and *m* − *k* tissues shared a different mechanism. For each setting, we evaluated 1,000 independent replicate test data sets, with tissue model parameters drawn from the same wide distributions as in the training data set.

Comparisons among these diverse scenarios illustrates that the classification performance of CLOUD is dependent on a combination of the difference between flexibilities in model parameters of the two retention mechanisms and the numbers of tissues sharing each retention mechanism (supplementary fig. S6, Supplementary Material online). In particular, the retention mechanism with greater flexibility in model parameters is most frequently chosen by CLOUD unless a majority of tissues share the more constrained retention mechanism. For example, when Tissue Mechanism A is “Conserved” (the most constrained retention mechanism) and shared by k∈{1,2,3,4,5} tissues, and Tissue Mechanism B is any other (more flexible) retention mechanism and shared by 6−k tissues, Tissue Mechanism B is most frequently chosen by CLOUD unless the “Conserved” retention mechanism is shared by the majority (either four or five) of the tissues. Moreover, an intriguing pattern emerges when both retention mechanisms have the same flexibility, but differ in directionality of expression divergence, that is, when one is “Neofunctionalized parent” and the other is “Neofunctionalized child.” In such cases, one of these mechanisms is still chosen most often when it is shared by five of the six tissues. However, for all other scenarios, “Specialized” is the most prominently inferred retention mechanism. This result is sensible, as “Neofunctionalized parent” is characterized by a change in the parent copy, “Neofunctionalized child” by a change in the child copy, and “Specialized” by unconstrained changes in both parent and child copies. In summary, if two different retention mechanisms are each shared by a subset of the tissues, then the more flexible retention mechanism will be predominantly chosen unless the more constrained retention mechanism is shared by the majority of the tissues.

Despite its difficulty in classifying retention mechanisms under these mixed scenarios, CLOUD is generally unbiased in its parameter predictions (supplementary figs. S7–S11, Supplementary Material online). Though mixtures of retention mechanisms occasionally lead to biased parameter predictions, such as the overestimation of $\sigma^{2}$ for the setting of one “Conserved” tissue and five “Specialized” tissues (supplementary fig. S7, Supplementary Material online), or the underestimation of *α* for the setting of three “Conserved” and “Subfunctionalized” tissues (supplementary fig. S9, Supplementary Material online), estimates of expression states ($\theta_{P},\;\theta_{C}$, and $\theta_{A}$) are always unbiased. Thus, the parameters supporting specific retention mechanisms are well-estimated on average (supplementary figs. S7–S11, Supplementary Material online). These results highlight that under complex settings, CLOUD should accurately predict underlying evolutionary parameters, even in situations for which no single classified retention mechanism is correct or sensible (supplementary fig. S6, Supplementary Material online).

### Application of CLOUD to Empirical Data from *Drosophila*

Our simulation experiments highlight the exceptional classification performance of CLOUD relative to CDROM, as well as the unique ability of CLOUD to predict parameters underlying the evolution of duplicate genes. Hence, we next sought to use CLOUD to classify retention mechanisms and predict parameters of 208 duplicate genes in *Drosophila* (Assis and Bachtrog 2013) from their expression data in six tissues (Assis 2019b). Specifically, we first used PhyML (Guindon et al. 2010) to estimate a gene tree relating each parent, child, and ancestral gene in this data set of duplicate genes (Assis and Bachtrog 2013) (see Materials and Methods section). Next, as in our simulation studies, we trained CLOUD (assuming two hidden layers) on a large balanced simulated training set of 50,000 observations (10,000 from each of five classes), with evolutionary parameters $\theta_{P},\theta_{C},\theta_{A}\in [-4,4],\;\;{\text{log}}_{10}(\alpha )\in [0,3]$, and $\;{\text{log}}_{10}(\sigma^{2})\in [-2,3]$ drawn independently across six tissues, for a total of 30 random parameters per simulated training observation (see Materials and Methods section). We tailored CLOUD to this data set of duplicate genes (Assis and Bachtrog 2013) by generating *p *=* *108 input features (table 1) from comparisons to the empirical distribution of single-copy genes identified in this lineage (Assis and Bachtrog 2013) (see Materials and Methods section). Then, we used CLOUD to classify retention mechanisms and predict parameters of the 208 *Drosophila* duplicate genes (Assis and Bachtrog 2013) from these features.

**Fig. 5:**
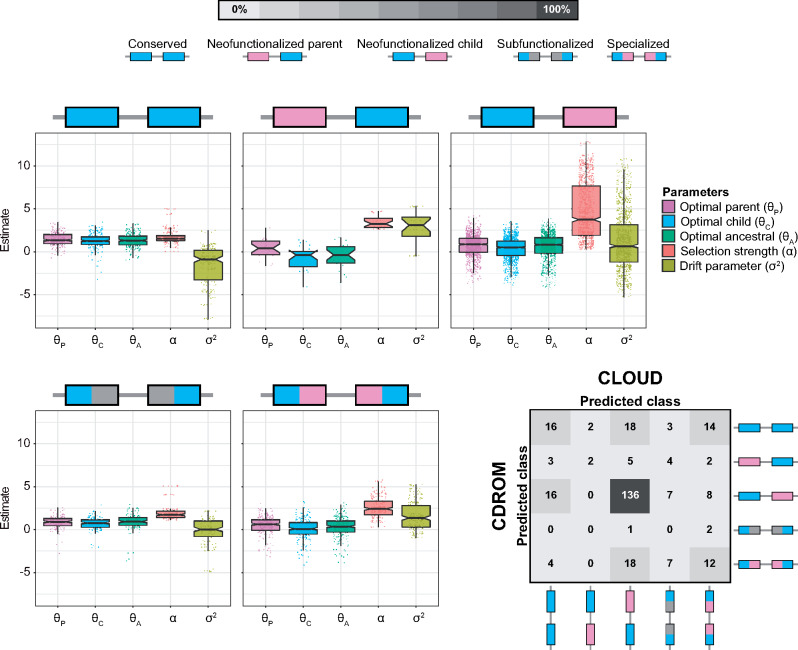
Classification and prediction results for application of CLOUD with *L *=* *2 hidden layers to empirical data from *Drosophila* (Assis and Bachtrog 2013; Assis 2019b). Box plots overlaid onto strip plots show distributions of log-transformed parameter estimates for each of the five classes of duplicate gene retention mechanisms. Note that six estimates, corresponding to the six tissues in the empirical data set, are plotted for each parameter. The confusion matrix in the bottom right illustrates the high concordance in classifications of CLOUD and CDROM for these empirical data, with both methods classifying the majority of duplicate genes as retained by neofunctionalization of the child copy.

Analysis of the resulting classifications reveals that the predominant mechanism of duplicate gene retention in *Drosophila* is neofunctionalization in which the child copy acquires a new function (61.43%; fig. 5), mirroring the findings of Assis and Bachtrog (2013). Moreover, classifications of CLOUD are generally concordant with those of CDROM (59.29%), with three key differences. In particular, of the 167 duplicates classified as “Neofunctionalized child” by CDROM, 16 are classified as “Conserved” by CLOUD. In addition, of the 53 duplicates classified as “Conserved” by CDROM, 18 are classified as “Neofunctionalized child” and 14 as “Specialized” by CLOUD. Finally, of the 41 duplicates classified as “Specialized” by CDROM, 18 are classified as “Neofunctionalzied child” by CLOUD. Based on our simulation results (supplementary figs. S1 and S2, Supplementary Material online), it is likely that these discrepancies reflect differences in the abilities of the CLOUD and CDROM classifiers to handle gene expression stochasticity.

We next examined the parameter predictions of CLOUD. Here, our major question was whether these predictions match theoretical expectations of duplicate gene retention mechanisms. To answer this question, we examined distributions of empirical parameter estimates for each class obtained with the CLOUD classifier (fig. 5). We first considered optimal expression estimates $\theta_{P},\;\theta_{C}$, and $\theta_{A}$. For the “Conserved” class, the distributions of estimated $\theta_{P},\;\theta_{C}$, and $\theta_{A}$ are not significantly different from one another, consistent with expectations (table 2). For the “Neofunctionalized parent” class, the distribution of $\theta_{P}$ is different (though not significantly) from those of $\theta_{C}$ and $\theta_{A}$, whereas the distributions of $\theta_{C}$ and $\theta_{A}$ are not significantly different from one another. This qualitative pattern is as expected (table 2), with the lack of a significant difference of $\theta_{P}$ likely due to the small number of duplicate genes in this class. For the “Neofuntionalized child” class, the distribution of $\theta_{C}$ is significantly different from those of $\theta_{P}$ and $\theta_{A}$, whereas the distributions of $\theta_{P}$ and $\theta_{C}$ are not significantly different from one another, also consistent with theoretical expectations (table 2). It is also interesting that, relative to other values of *θ*, $\theta_{P}$ is increased in the “Neofunctionalized parent” class, whereas $\theta_{C}$ is decreased in the “Neofunctionalized child” class. Though the sample size for the “Neofunctionalized parent” class is small, decreased $\theta_{C}$ in the “Neofunctionalized child” class is consistent with lower gene expression levels of testis-specific genes (Brawand et al. 2011; Assis and Bachtrog 2013), which compose a majority of the child duplicate gene copies in our data set (Assis and Bachtrog 2013). For the “Subfuntionalized” class, distributions of $\theta_{P}$ and $\theta_{C}$ are different (though not significantly) from one another, with the center of the distributions of $\theta_{P}$ and $\theta_{C}$ located at approximately the center of the $\theta_{A}$ distribution. This qualitative pattern matches expectations, though formal tests of significance were again underpowered due to only a handful of duplicates classified as “Subfunctionalized.” Finally, for the “Specialized” class, $\theta_{P},\;\theta_{C}$, and $\theta_{A}$ are all significantly different from one another, matching theoretical expectations (table 2). Analogously, we also observe a general concordance between estimates of *α* and $\sigma^{2}$ and theoretical expectations of classified duplicate gene retention mechanisms. In particular, duplicate genes classified as neofunctionalized or specialized have significantly elevated estimated selection strengths (*α*) compared to those classified as conserved or subfunctionalized (fig. 5). These differences are consistent with theoretical expectations, as both neofunctionalization and specialization result in acquisitions of new functions that are hypothesized to be driven by strong selection, whereas both conservation and subfunctionalization result in preservations of ancestral functions that may occur in the absence of selection. Further, estimates of phenotypic drift ($\sigma^{2}$) are also significantly larger for duplicate genes classified as neofunctionalized or specialized than as conserved or subfunctionalized. This result supports the hypothesis that traits require some minimum threshold of plasticity to effectively explore the space of novel states on which selection may act.

As a final analysis of the empirical data, we performed a case study of the child gene *Dntf-2r* and its parent *Dntf-2*. We chose this duplication event because it was well-characterized in earlier studies (Betrán and Long 2003; Assis and Bachtrog 2013; Jiang and Assis 2017), providing us with a baseline for comparing our findings. In particular, *Dntf-2r* arose in the *D. melanogaster* lineage after its divergence from the *D. pseudoobscura* lineage (Betrán and Long 2003; Assis and Bachtrog 2013). Several studies showed that *Dntf-2r* is specifically expressed in the testis and evolving under positive selection, whereas its parent *Dntf-2* is expressed broadly across tissues and evolving under negative selection (Bhattacharya and Steward 2002; Betrán and Long 2003; Assis and Bachtrog 2013; Jiang and Assis 2017). Hence, it has been hypothesized that *Dntf-2r* underwent neofunctionalization and acquired a new male-specific function after duplication (Betrán and Long 2003; Assis and Bachtrog 2013). Consistent with this hypothesis, *Dntf-2r* and *Dntf-2* are classified by both CDROM and CLOUD as retained by neofunctionalization of the child copy. Moreover, CLOUD estimates mean $\;{\text{log}}_{10}(\alpha )=4.254$ and mean $\;{\text{log}}_{10}(\sigma^{2})=-3.429$, supporting previous findings that strong selection and weak phenotypic drift underlie neofunctionalization of *Dntf-2r* (Betrán and Long 2003; Jiang and Assis 2017).

## Discussion

In this study, we have demonstrated that modeling of expression evolution and application of modern statistical learning techniques substantially enhances performance in classifying the retention mechanisms of duplicate genes and predicting their underlying parameters. Specifically, our new method CLOUD has high power and accuracy in discriminating among five classes of duplicate gene retention mechanisms (fig. 3, supplementary figs. S1–S4, Supplementary Material online), and high accuracy in parameter estimation (fig. 4). It represents a major advancement over the only previously available expression-based method, CDROM (Assis and Bachtrog 2013; Perry and Assis 2016), which has much lower classification power and accuracy (fig. 3), displays strong classification bias (supplementary figs. S1 and S2, Supplementary Material online), and cannot perform the task of parameter prediction at all. Thus, CLOUD represents a major advancement in classifying duplicate gene retention mechanisms and predicting their evolutionary parameters. Moreover, though our study focuses on its application to gene expression data from multiple tissues, CLOUD can also be applied to gene expression data from multiple conditions of different types (e.g., developmental stages or disease states), or even to gene expression data from a single condition, which is always the case for single-celled organisms. As a result, CLOUD can be used to learn about evolution after gene duplication in many diverse biological systems.

When designing the multi-layer neural network architecture of CLOUD, we took measures to mitigate overfitting through elastic net-style regularization (Zou and Hastie 2005), which shrinks model weights through a mixture of *L*_1_- and *L*_2_-norm penalties (Hastie et al. 2009). However, several other approaches, such as early stopping (Bishop 1995; Sjöberg and Ljung 1995; Goodfellow et al. 2016) and dropout (Srivastava et al. 2014; Goodfellow et al. 2016), could have been used instead to achieve a similar goal. Of the two alternatives mentioned, the dropout regularization procedure is closer to our approach, with a key difference in that regularization proceeds in a more stochastic fashion. Specifically, regularization is performed by dropping some proportion x∈(0,1) of hidden units uniformly at random in each layer during each training epoch, thereby ensuring that fewer model parameters (weights) are trained during each round of training. The optimal proportion *x* would then be chosen through cross-validation (Hastie et al. 2009), with all hidden units subsequently used during testing. Another option for reducing overfitting is ensembling (Breiman 1996; Freund and Shapire 1996a, 1996b; Ridgeway 1999) of neural networks (of which dropout is a specific form), either through bagging or boosting across neural networks (Schwenk and Bengio 1998; Goodfellow et al. 2016), or by boosting across hidden layers of a neural network (Bengio et al. 2006). Other ensemble approaches, such as random forests (Breiman 2001; Hastie et al. 2009) and boosted regression and classification trees (Ridgeway 1999; Hastie et al. 2009) may represent complementary flexible alternative frameworks to the neural network procedure employed here. In particular, they may be beneficial if expression data were absent for some tissues or genes, as they are able to naturally handle missing data (Hastie et al. 2009). Though we considered these other regularization forms, we chose to utilize elastic net-style regularization as we felt that it provided a natural and deterministic mechanism for controlling model complexity.

We also considered an alternative approach for constructing the CLOUD classifier and predictor by employing maximum likelihood estimation (Casella and Berger 2002; Brawand et al. 2011; Clavel et al. 2015). Specifically, given expression data for parent, child, and ancestral genes, one can use maximum likelihood to estimate the set of parameters $\left\{\theta_{P},\theta_{C},\theta_{A},\alpha ,\sigma^{2}\right\}$ from an OU model of expression evolution for each of the five retention mechanism classes, where optimal expression states ($\theta_{P},\theta_{C},\theta_{A}$) are constrained as shown in table 2. Then, one can utilize likelihood ratio tests between models to derive a decision tree (similar to that used by CDROM) for performing classification. For these tests, the “Conserved” class would be nested within the “Neofunctionalized parent” and “Neofunctionalized child” classes, “Neofuntionalized parent” and “Neofunctionalized child” classes would be nested within the “Subfunctionalized” class, and the “Subfunctionalzied” class would be nested within the “Specialized” class. This procedure would require that model selection is performed using appropriate significance cutoffs (Casella and Berger 2002), accounting for multiple testing (Neyman and Pearson 1928). Furthermore, the fit of the likelihood model would be highly dependent on underlying assumptions (e.g., independence among tissues and gene tree estimates), and the “Specialized” model with five free parameters per tissue (table 2) would be over-parameterized without including expression data from a fourth gene in an outgroup species. Additionally, it would be difficult to directly incorporate the genome-wide distribution of expression differences at single-copy genes to use as a baseline level of expression divergence. For these reasons, we believe that the framework implemented by CLOUD represents a more appropriate, powerful, and flexible approach for learning evolutionary retention mechanisms and parameters of duplicate genes.

Application of CLOUD to empirical data from *Drosophila* (Assis and Bachtrog 2013; Assis 2019b) recapitulated many of the classifications previously inferred by CDROM (Assis and Bachtrog 2013), notably classifying the majority of duplicate genes as retained by neofunctionalization in which the child copy acquires a new function (fig. 5). Predicted parameters of *Drosophila* duplicate genes were also generally concordant with theoretical expectations of their classified retention mechanisms (table 2, fig. 5). In particular, observed differences among distributions of optimal expression estimates for parent ($\theta_{P}$), child ($\theta_{C}$), and ancestral ($\theta_{A}$) genes matched expectations for all retention mechanism classes (table 2, fig. 5). Similarly, distributions of selection strength (*α*) estimates were shifted toward higher values for retention mechanisms in which there were acquisitions of new functions (neofunctionalization and specialization) relative to those in which ancestral functions were preserved (conservation and subfunctionalization, fig. 5), consistent with hypotheses that strong positive selection drives fixation of new functions after gene duplication (Ohno 1970; He and Zhang 2005; Rastogi and Liberles 2005). Interestingly, distributions of phenotypic drift ($\sigma^{2}$) estimates were also elevated for classes in which there were acquisitions of new functions (fig. 5), perhaps because increased levels of plasticity are necessary to explore new functions on which selection can act. This hypothesis is also supported by other studies of these *Drosophila* duplicate genes (Assis and Bachtrog 2013), which found evidence of parallel sequence and expression evolution for all classified retention mechanisms (Assis and Bachtrog 2013; Jiang and Assis 2017). Thus, our empirical findings are largely consistent both with long-held theoretical predictions (Ohno 1970; He and Zhang 2005; Rastogi and Liberles 2005), and with results from previous analyses of these *Drosophila* duplicate genes (Assis and Bachtrog 2013; Jiang and Assis 2017). Taken together, they illustrate that functional divergence after gene duplication in *Drosophila* is often asymmetric, tends to affect the child copy, and is driven by strong selection.

## Materials and Methods

In this section, we detail the algorithmic choices used to train CLOUD, the simulation setting used to compare its performance to the classifier CDROM, and the necessary steps for application of CLOUD and CDROM to empirical data from *Drosophila*.

### Training the Neural Network on Data Simulated from OU Processes

Consider a set of *N_k_* training observations for class k∈{1,2,3,4,5}, such that the total training sample size is $N=N_{1}+N_{2}+N_{3}+N_{4}+N_{5}$. For observation i∈{1,2,…,N} and output k∈{1,2,…,K}, let $y_{k}^{\left(i\right)}$ denote the true value and ${\hat{y}}_{k}^{\left(i\right)}$ denote the estimated value. We wish to train a neural network model to minimize the overall discrepancy between $y_{k}^{\left(i\right)}$ and ${\hat{y}}_{k}^{\left(i\right)}$, which we measure with the loss function $ℒ({\hat{y}}^{\left(i\right)},y^{\left(i\right)})$, across the *N* samples and *K* outputs. Let

$$W^{\left[\ell \right]}=\left[\begin{array}{cccc} w_{11}^{\left[\ell \right]} & w_{12}^{\left[\ell \right]} & \cdots & w_{2p[\ell +1]}^{\left[\ell \right]}\\ w_{21}^{\left[\ell \right]} & w_{22}^{\left[\ell \right]} & \cdots & w_{2p[\ell +1]}^{\left[\ell \right]}\\ \vdots & \vdots & \ddots & \vdots \\ w_{p[\ell ]1}^{\left[\ell \right]} & w_{p[\ell ]2}^{\left[\ell \right]} & \cdots & w_{p[\ell ]p[\ell +1]}^{\left[\ell \right]} \end{array}\right]\in {\mathbb{R}}^{p[\ell ]\times p[\ell +1]}$$

be the matrix of weights going from layer ℓ to layer ℓ+1 for ℓ∈{0,1,…,L}, and let

$$w=\left(w_{0}^{\left[0\right]},w_{0}^{\left[1\right]},\ldots ,w_{0}^{\left[L\right]}\right)\in {\mathbb{R}}^{L+1}$$

denote the vector of biases for all of the layers.

To train the neural network, we wish to identify the set of parameters (weights and biases) $W=\{w,W^{\left[0\right]},\ldots ,W^{\left[L\right]}\}$ that minimize the cost

$$J(W,L)=\frac{1}{N}\sum_{i=1}^{N}ℒ({\hat{y}}^{\left(i\right)},y^{\left(i\right)}).$$

To prevent overfitting, we include an elastic net-style regularization penalty term (Zou and Hastie 2005) on the weights of each layer with two tuning hyper parameters. Specifically, we reduce the complexity of the fitted model with the tuning parameter λ≥0, which shrinks the magnitude of the weights to zero. We also perform simultaneous weight shrinkage and feature selection with the elastic net tuning parameter γ∈[0,1], such that we are performing *L*_2_-norm regularization when *γ*  =  0, *L*_1_-norm regularization that incorporates feature selection when *γ* = 1, and both types of regularization when γ∈(0,1). In particular, we seek to find the model parameters W that minimize the penalized cost function

$$J(W,L,\lambda ,\gamma )=\frac{1}{N}\sum_{i=1}^{N}ℒ\left({\hat{y}}^{\left(i\right)},y^{\left(i\right)}\right)+\lambda \sum_{\ell =0}^{L}\sum_{j=1}^{p[\ell ]}\sum_{k=1}^{p[\ell +1]}\left[\left(1-\gamma ){\left(w_{jk}^{\left[\ell \right]}\right)}^{2}+\gamma |w_{jk}^{\left[\ell \right]}\right|\right].$$

In the classification problem, for training observation i∈{1,2,…,N}, we define the indicator random variable $y_{k}^{\left(i\right)}=1$ if observation *i* is from class *k*, and 0 otherwise. Hence, all output values are zero except for that corresponding to class *k*, which has a value of one. Based on this output, we employ the loss function used in J(W,L,λ,γ) as the categorical cross entropy deviance (Goodfellow et al. 2016)

$$ℒ\left({\hat{y}}^{\left(i\right)},y^{\left(i\right)}\right)=-\sum_{k=1}^{K}y_{k}^{\left(i\right)}\;\text{log}\;\left({\hat{y}}_{k}^{\left(i\right)}\right).$$

In the prediction problem, $y_{k}^{\left(i\right)}$ is instead the *k*th parameter value from simulated replicate *i*. Based on this output, we employ the loss function used in J(W,L,λ,γ) as the residual sum of squared error

$$ℒ\left({\hat{y}}^{\left(i\right)},y^{\left(i\right)}\right)=\sum_{k=1}^{K}{\left({\hat{y}}_{k}^{\left(i\right)}-y_{k}^{\left(i\right)}\right)}^{2}.$$

Simulated data have been successfully used to train models for learning about evolution from genomic data in many recent studies (Lin et al. 2011; Schrider and Kern 2016; Sheehan and Song 2016; Kern and Schrider 2018; Schrider et al. 2018; Sugden et al. 2018; Flagel et al. 2019; Mughal et al. 2020; Mughal and DeGiorgio 2019; Adrion et al. 2020). Therefore, to train CLOUD for both classification and prediction, we generated a balanced simulated data set with 10^4^ observations from each of the five classes, for a total of *N *=* *50,000 training observations. We assumed that tissues were independent, and that there were a total of *m *=* *6 tissues as in an empirical data from *Drosophila* (Assis 2019b) that we later applied our method to, for a total of *p *=* *108 input features.

To make the simulated data set more realistic, we drew model parameters $T_{\text{PC}}$ and $T_{\text{PCA}}$ from empirical gene tree estimates for the set of *Drosophila* duplicate genes used by Assis and Bachtrog (2013). The procedure for estimating these gene trees is detailed in subsection *Application of CDROM and CLOUD to empirical data from Drosophila* below. For all analyses, we scaled the root of the gene tree to have height one, and considered a new scaled time for the duplication event of $t_{\text{PC}}=T_{\text{PC}}/T_{\text{PCA}}$, such that $t_{\text{PC}}$ represented the time of the duplication relative to the height of the root of the gene tree. For a given class, we drew parameters $\Omega ={\left\{t_{\text{PC}},\theta_{Pj},\theta_{Cj},\theta_{Aj},\alpha_{j},\sigma_{j}^{2}\right\}}_{j=1}^{m}$ uniformly at random, assuming that $\theta_{\text{PC}j}=\theta_{\text{PCA}j}=\theta_{Aj}$ for tissue *j* (schematic provided in fig. 2*D*). In particular, we drew $t_{\text{PC}}$ from the distribution of empirical gene tree estimates, $\theta_{j}\in [-4,4]$ for j∈{P,C,A}, *α* from $\;{\text{log}}_{10}(\alpha )\in [0,3]$, and $\sigma^{2}$ from $\;{\text{log}}_{10}(\sigma^{2})\in [-2,3]$. We chose this specific range for $\theta_{P},\;\theta_{C}$, and $\theta_{A}$ because we found that differences in $\;{\text{log}}_{10}$-transformed empirical expression data were normally distributed, matching expectations under an OU model. For this reason, all empirical expression data were also $\;{\text{log}}_{10}$-transformed prior to applying CLOUD. The class *k* is determined by $\left\{\theta_{P},\theta_{C},\theta_{A}\right\}$, which is summarized in table 2. Then, we simulated gene expression data $e^{\left(i\right)}\in {\mathbb{R}}^{3m}$ under model parameters for a given class *k* (table 2), assuming independence among tissues, and generated *N_k_* simulated replicates of parameter values $\Omega_{k}^{\left(i\right)}$ for $i=1,2,\ldots ,N_{k}$.

Given a number of hidden layers *L* and the pair of regularization tuning parameters *λ* and *γ*, we estimated the set of parameters W using the Adam optimizer (Kingma and Ba 2014) with learning rate $10^{-3}$ and exponential decay rates for the first and second moment estimates of $\beta_{1}=0.9$ and $\beta_{2}=0.999$ (Kingma and Ba 2014), respectively. This optimizer was used as it efficiently traverses the cost function surface J(W,L,λ,γ) to rapidly identify the minimum (Kingma and Ba 2014). We also used mini-batch optimization with a batch size of 5,000 observations for 500 epochs. To estimate *L*, *λ*, and *γ*, we performed five-fold cross-validation (Hastie et al. 2009). Specifically, we used 80% (40,000) of observations for training, and held out the remaining 20% (10,000) for validation. We also ensured that the training and validation sets were balanced in class representation, such that there were equal numbers of observations from each class in the training (8,000) and validation (2,000) sets. To assess method performance for a given fold, we computed the validation loss

$$\text{Validation}\;\text{loss}=\frac{1}{2000}\sum_{i\in \text{Validation}\;\text{set}}ℒ\left({\hat{y}}^{\left(i\right)},y^{\left(i\right)}\right),$$

where the loss is either the categorical cross entropy deviance or the residual sum of squared error for the classifier or predictor, respectively (Goodfellow et al. 2016). We then averaged this validation loss across the five folds to compute the cross-validation error (Hastie et al. 2009). We considered values of L∈{0,1,2,3} and γ∈{0,0,0.1,…,1.0}, as well as 25 values of *λ* chosen uniformly across $\;{\text{log}}_{10}(\lambda )\in [-12,-3]$. Given the optimal cross-validation estimates $\hat{L},\;\hat{\lambda }$, and $\hat{\gamma }$ for *L*, *λ*, and *γ*, respectively, we estimated the neural network model parameters $W=\{w,W^{\left[0\right]},\ldots ,W^{\left[\hat{L}\right]}\}$ as

$$\hat{W}=\begin{array}{c} \text{arg}\;\text{min}\\ W \end{array}J(W,\hat{L},\hat{\lambda },\hat{\gamma }).$$

Previous studies have found that neural networks with enough hidden layers or units can approximate any function, and therefore lead to overfitting (Cybenko 1989; Goodfellow et al. 2016). Hence, based on simulations, we estimated that a neural network with $\hat{L}=2$ hidden layers provides the best cross-validation performance, with the validation loss for the classifier of approximately 0.918 with optimal tuning parameters $\hat{\lambda }\approx 1.778\times 10^{-4}$ and $\hat{\gamma }=1$, and the validation loss for the predictor of approximately 0.899 with optimal tuning parameters $\hat{\lambda }\approx 7.499\times 10^{-8}$ and $\hat{\gamma }=0.8$. Comparisons of classification and prediction performances across the four network architectures L∈{0,1,2,3} are given in supplementary figures S12 and S13, Supplementary Material online, highlighting the generally superior performance of the architecture with two hidden layers.

### Application of CDROM and CLOUD to Simulated Test Data

To compare the relative classification powers and accuracies of CDROM and CLOUD and explore the prediction accuracy of CLOUD, we simulated training and test data sets for duplicate genes based on an OU process, which is described in subsection *Training the neural network on data simulated from OU processes* above. However, in that subsection, we assumed that expression vectors for single-copy genes G were given. These would typically be extracted from the genome-wide distribution of single-copy genes for the pair of species being studied, such that trained models are based on the level of expression divergence typically observed in the study system.

For our simulated training and test sets, we generated a background set of 10,000 six-tissue expression vectors for single-copy genes that was inspired by those of the single-copy genes identified in *Drosophila* (Assis and Bachtrog 2013; Assis 2019b). Specifically, we applied the Brownian motion model (Felsenstein 1973) implemented in mvmorph (Clavel et al. 2015) to expression vectors of single-copy genes between Species 1 and 2, assuming that tissues were independent and that the root of the two-species phylogeny had height one, to estimate ancestral expression *θ* and variance $\sigma^{2}$ parameters consistent with the empirical distribution in *Drosophila* at each tissue and single-copy gene. Given the set of parameters, we then sampled values of *θ* and $\sigma^{2}$ uniformly at random from the estimated empirical distribution, and generated simulated single-copy expression vectors in Species 1 and Species 2 for *m *=* *6 independent tissues, giving us the simulated set G.

To test either the classifier or predictor, we generated a balanced set of duplicate gene expression vectors, such that each of the five classes had 1,000 observations, for a total of *N *=* *5,000 test observations. We assumed that tissues were independent, and that there was a total of *m *=* *6 tissues as in the training set. For a given class, we drew parameters $\Omega ={\left\{t_{\text{PC}},\theta_{Pj},\theta_{Cj},\theta_{Aj},\alpha_{j},\sigma_{j}^{2}\right\}}_{j=1}^{m}$ uniformly at random. In particular, as with the training set, we drew $t_{\text{PC}}$ from the distribution of empirical gene tree estimates, $\theta_{j}\in [-4,4]$ for j∈{P,C,A}, *α* from $\;{\text{log}}_{10}(\alpha )\in [0,3]$, and $\sigma^{2}$ from $\;{\text{log}}_{10}(\sigma^{2})\in [-2,3]$. The class *k* was determined by $\left\{\theta_{P},\theta_{C},\theta_{A}\right\}$ (table 2), and gene expression data were generated $e^{\left(i\right)}\in {\mathbb{R}}^{3m}$ under model parameters for a given class *k* (table 2), assuming independence among tissues and *N_k_* simulated replicates of parameter values $\Omega_{k}^{\left(i\right)}$ for $i=1,2,\ldots ,N_{k}$.

To assay how CLOUD performs in different portions of the parameter space, we also examined its accuracy on test sets drawn from restricted parameter values. Specifically we considered three distinct ranges for *α* of [1,10], [10,100], and[100, 1,000], and five distinct ranges for $\sigma^{2}$ of [0.01, 0.1], [0.1, 1], [1, 10], [10, 100], and [100, 1,000]. For each combination of a range for *α* and a range for $\sigma^{2}$, we sampled *α* and $\sigma^{2}$ uniformly at random.

### Application of CDROM and CLOUD to Empirical Data from *Drosophila*

We applied CDROM and CLOUD to empirical data consisting of *Drosophila* duplicate and single-copy genes (Assis and Bachtrog 2013) and their expression abundances in six tissues (Assis 2019b). In particular, duplicate and single-copy genes in *D. melanogaster* and *D. pseudoobscura* were obtained from Assis and Bachtrog (2013). In that study, pairs of duplicate genes in each species were identified via BLAST searches (Altschul et al. 1990) and supplemented with those from Chen et al. (2010). A table of orthologs, or genes that arose from the same common ancestor in 12 sequenced *Drosophila* species (Drosophila 12 Genomes Consortium 2007), was downloaded from FlyBase at https://www.flybase.org. Orthologs were used to determine presence or absence of duplicate gene copies in *D. melanogaster* and *D. pseudoobscura*. Gene duplication events that occurred after the divergence of the *D. melanogaster* and *D. pseudoobscura* lineages were defined as those for which one duplicate gene copy is present in both species (parent) and the second is only present in one species (child). Quantile-normalized gene expression abundances for carcass, female head, ovary, male head, testis, and accessory gland tissues in *D. melanogaster* and *D. pseudoobscura* were obtained from the Dryad data set associated with Assis (2019b) at https://doi.org/10.5061/dryad.742564m. In that study, paired-end RNA-sequencing reads were downloaded from modENCODE (Celniker et al. 2009) at https://www.modencode.com, and aligned to reference transcriptomes of each species with Bowtie 2 (Langmead et al. 2009). Abundances in fragments per kilobase of exon per million fragments mapped (FPKM, Trapnell et al. 2013) were calculated with eXpress (Roberts and Pachter 2013), quantile-normalized, and log-transformed. After examination of the distribution of these values, genes with little or no expression in all tissues were removed.

Because CLOUD requires estimates of $T_{\text{PC}}$ and $T_{\text{PCA}}$, we first generated multiple sequence alignments with MACSE (Ranwez et al. 2018), which accounts for underlying codon structure, and then inferred a gene tree with PhyML (Guindon et al. 2010) for each parent, child, and ancestral gene in the duplication data set (Assis and Bachtrog 2013). The empirical distributions of estimated $T_{\text{PC}}$ and $T_{\text{PCA}}$ across these gene trees were used as input to an OU process to generate a balanced training set with *N *=* *50,000 observations as described in subsection *Training the neural network on data simulated from OU processes* above. Gene expression data from single-copy genes in *Drosophila* (Assis and Bachtrog 2013; Assis 2019b) were used as the set G necessary for application of both CDROM and CLOUD. We trained a classifier and predictor for CLOUD assuming *L *=* *2 hidden layers, and through five-fold cross-validation (Hastie et al. 2009), estimating the regularization tuning parameters as $\hat{\lambda }\approx 1.778\times 10^{-4}$ and $\hat{\gamma }=0.9$ for the classifier and $\hat{\lambda }=10^{-6}$ and $\hat{\gamma }=0.5$ for the predictor.

## Supplementary Material

Supplementary data are available at *Molecular Biology and Evolution* online.

### Data Availability

The data underlying this article were derived from sources in the public domain: FlyBase at https://www.flybase.org, modENCODE at https://www.modencode.com, and Dryad at https://doi.org/10.5061/dryad.742564m.

## Supplementary Material

msaa267_Supplementary_Data
