# Supplementary material for: Learning Retention Mechanisms and Evolutionary Parameters of Duplicate Genes from Their Expression Data
Source: Mol Biol Evol. 2020 Oct 12;38(3):1209–24. doi: 10.1093/molbev/msaa267 (PMC7947822; doi:10.1093/molbev/msaa267)
Supplement: msaa267_Supplementary_Data [file msaa267_supplementary_data.zip › DeGiorgio_Assis_Supplement.pdf]

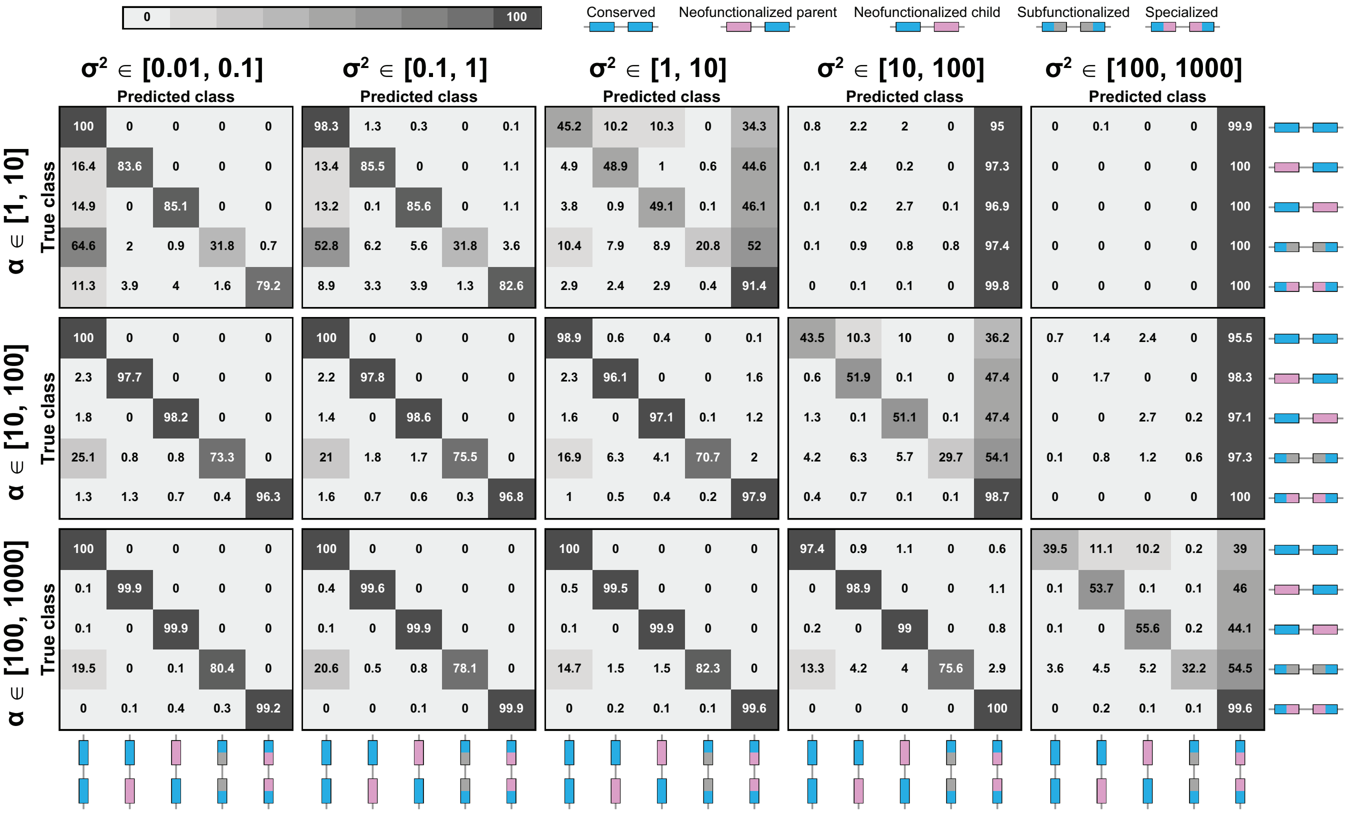

Figure S1: Confusion matrices for applications of `CDROM` to data simulated under specific parameter ranges for α and σ^2. Classification accuracy is highest for large α and small σ^2, and lowest for small α and large σ^2.

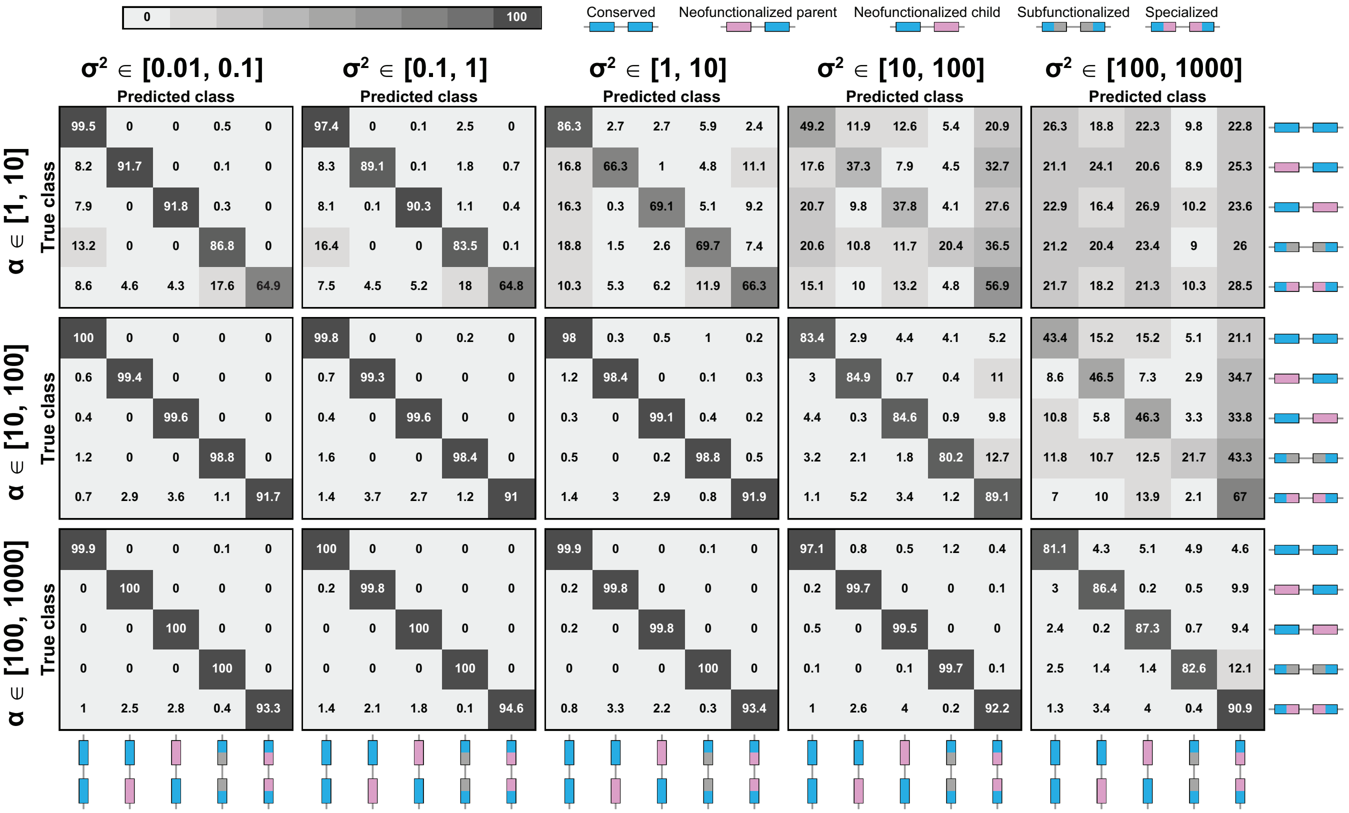

Figure S2: Confusion matrices for applications of `CLOUD` with $L = 2$ hidden layers to data simulated under specific parameter ranges for α and σ^2. Classification accuracy is highest for large α and small σ^2, and lowest for small α and large σ^2.

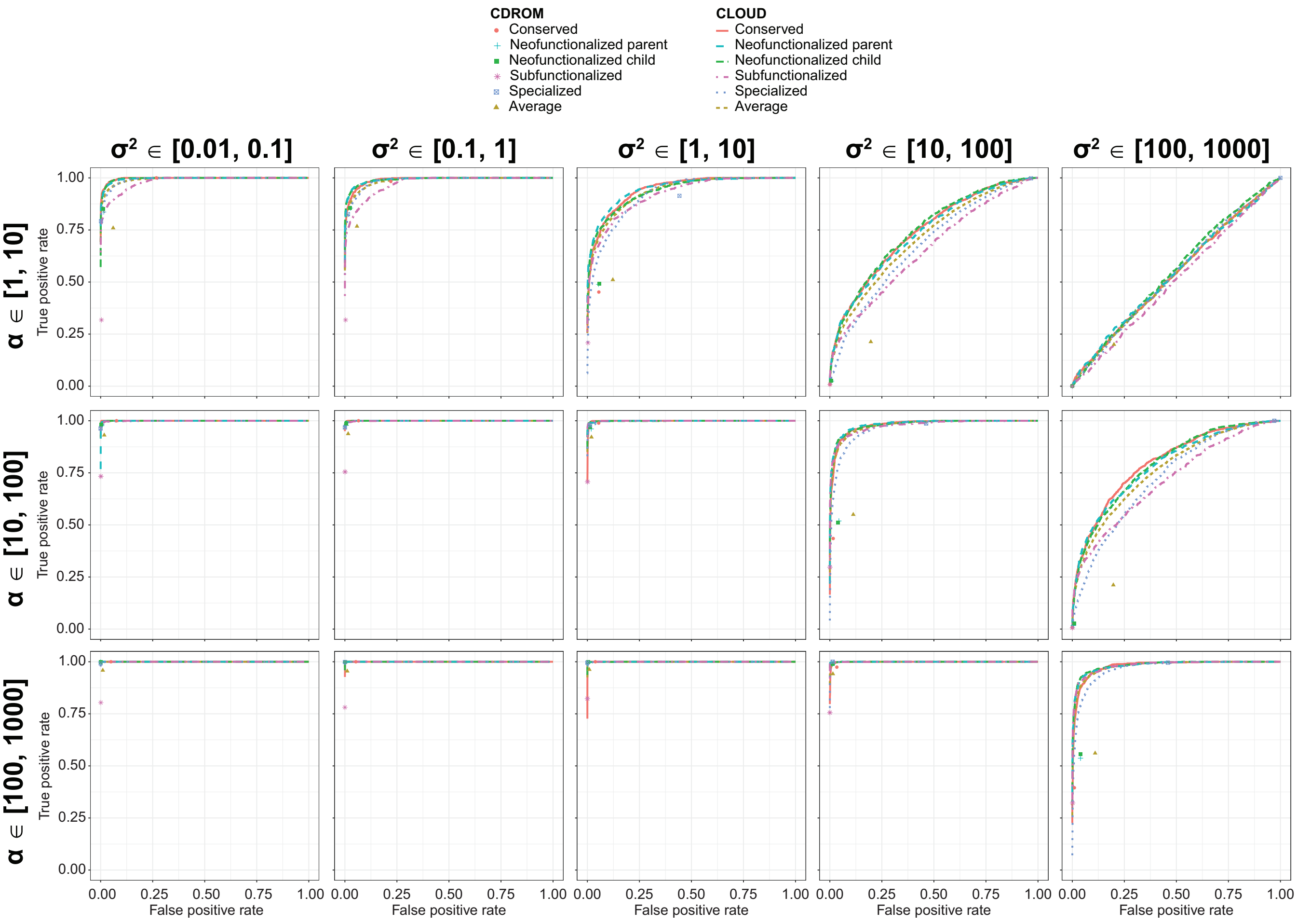

Figure S3: Receiver operating characteristic curves for applications of CDROM and CLOUD with $L = 2$ hidden layers to data simulated under specific parameter ranges for α and σ^2. Classification accuracy is highest for large α and small σ^2, and lowest for small α and large σ^2. Because CDROM is a decision tree classifier, its true positive and false positive rates are plotted as points.

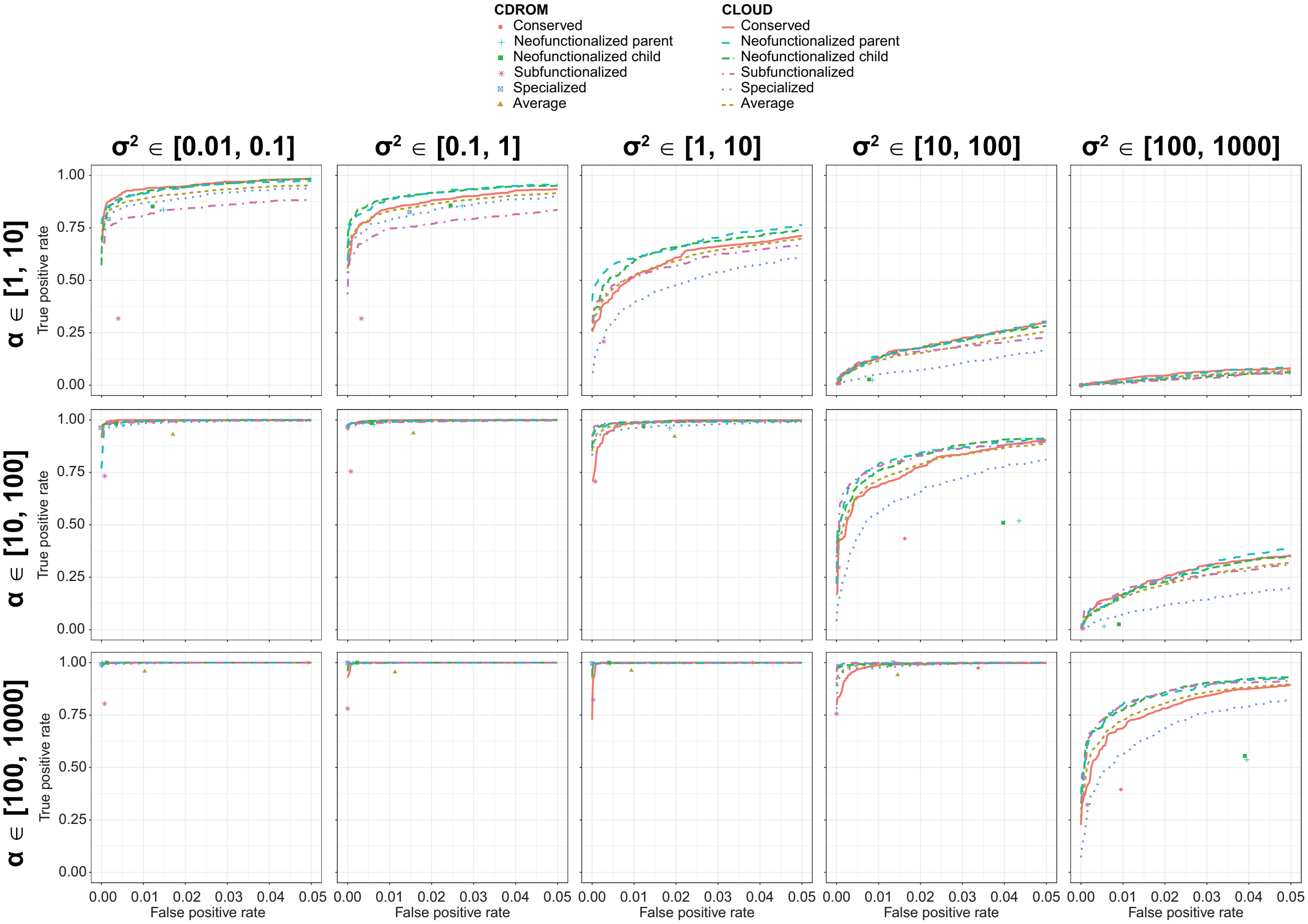

Figure S4: Receiver operating characteristic curves truncated at a false positive rate of 5% for applications of CDROM and CLOUD with $L = 2$ hidden layers to data simulated under specific parameter ranges for α and σ^2. Classification accuracy is highest for large α and small σ^2, and lowest for small α and large σ^2. Because CDROM is a decision tree classifier, its true positive and false positive rates are plotted as points.

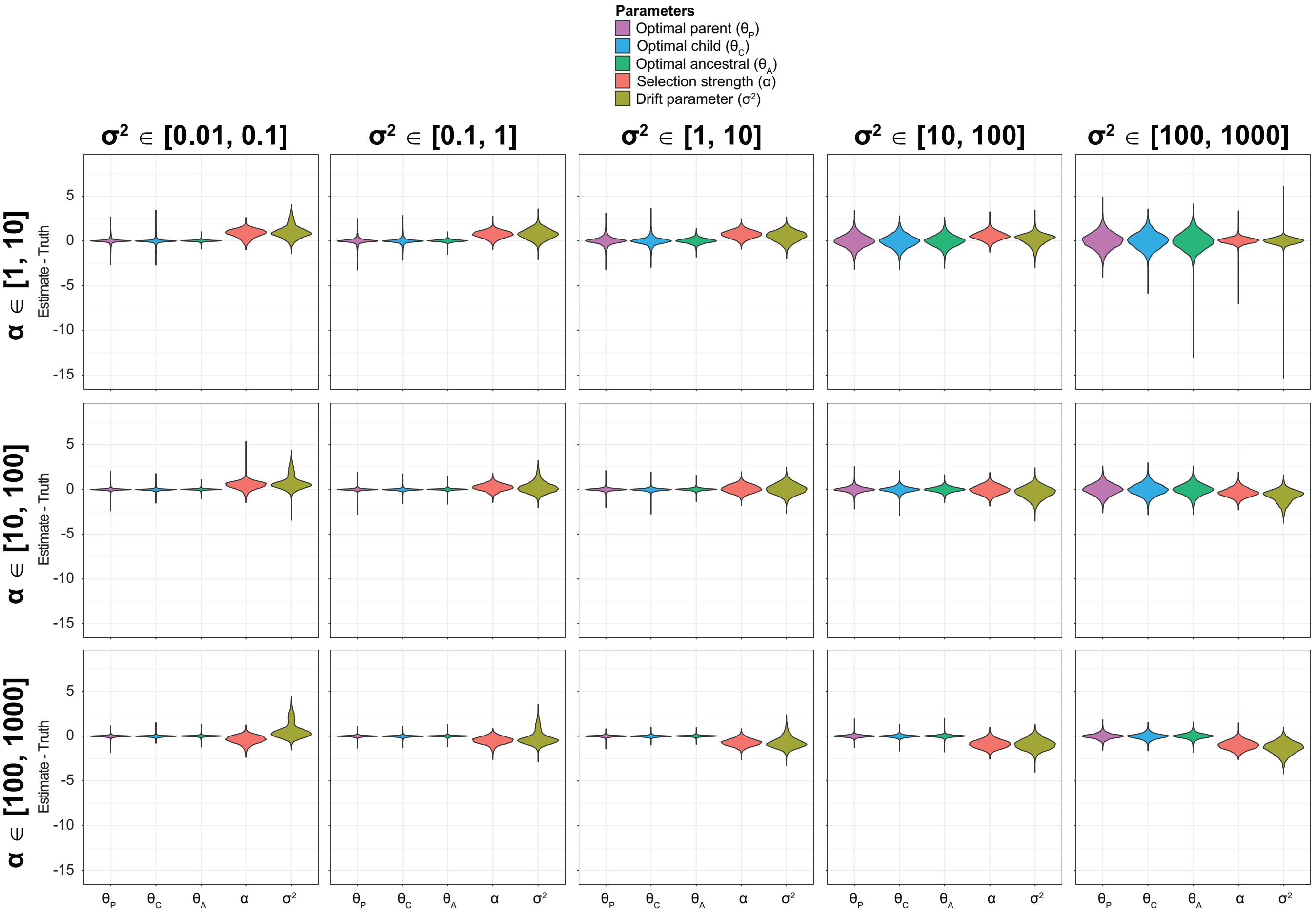

Figure S5: Prediction results for applications of `CLOUD` with $L = 2$ hidden layers to data simulated under specific parameter ranges for α and σ^2. Violin plots display distributions of mean parameter prediction errors across the $m = 6$ tissues for each simulated test set.

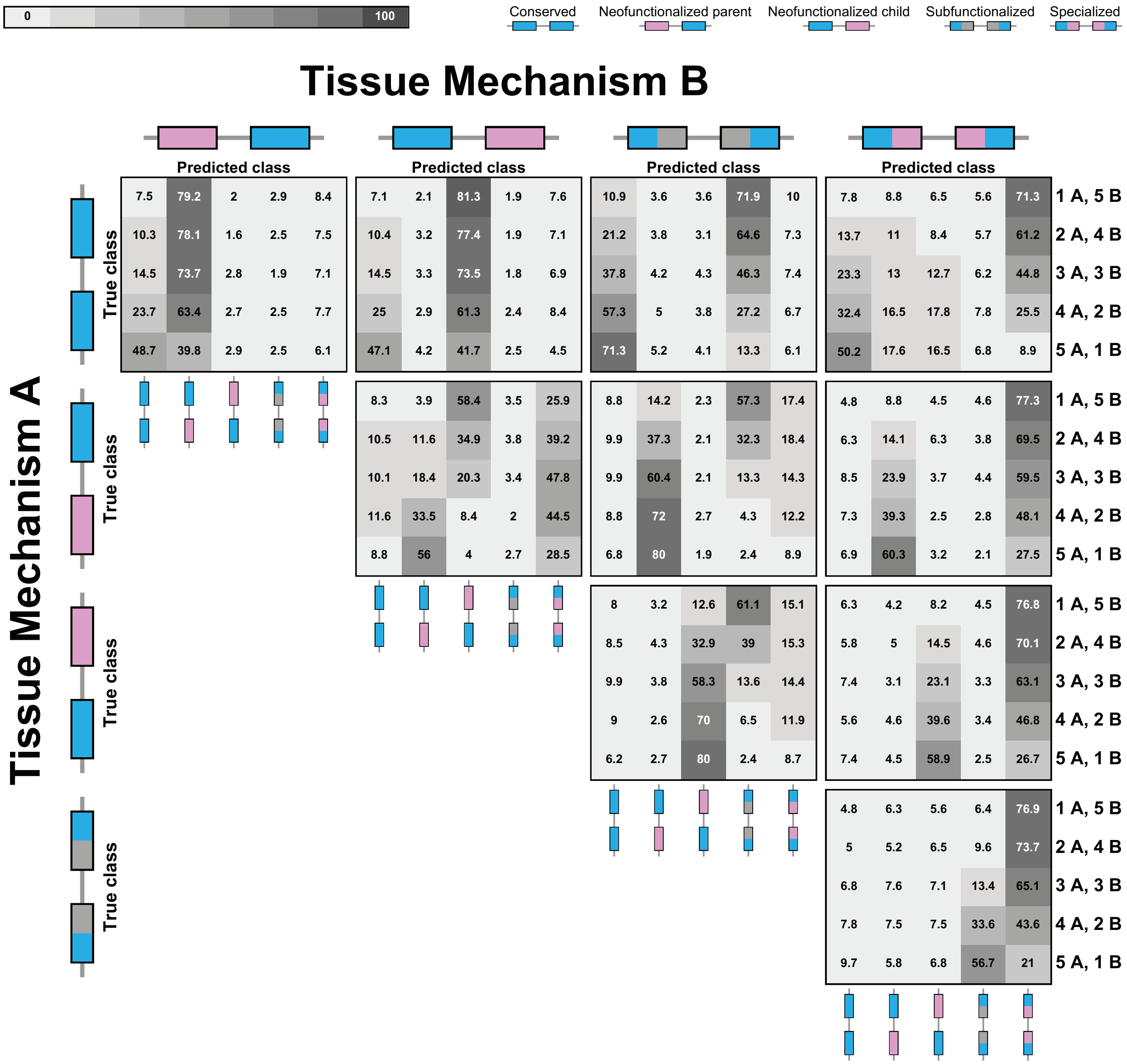

Figure S6: Confusion matrices for applications of CLOUD with $L = 2$ to data simulated under mixed retention mechanism scenarios. Each sub-panel represents a distinct pair of retention mechanisms, and all pairwise combinations of distinct retention mechanisms are considered. Within each confusion matrix, columns represent the five predicted retention mechanism classes of CLOUD, and rows represent the five settings for mixed retention mechanisms across tissues. Specifically, row $k \in \{1, 2, 3, 4, 5\}$ of a confusion matrix indicates that the test scenario includes k tissues sharing Tissue Mechanism A and $6 - k$ tissues sharing Tissue Mechanism B.

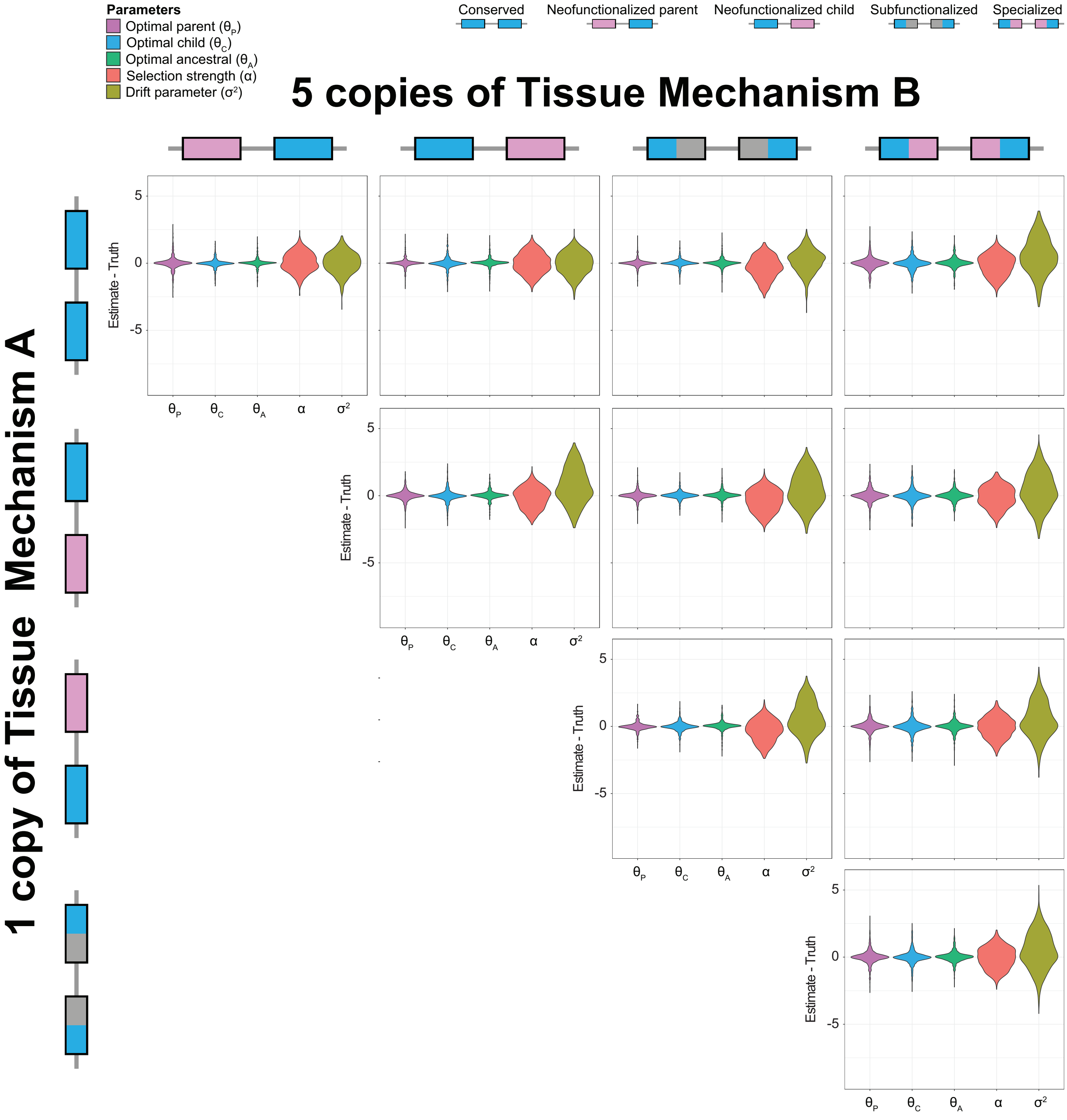

Figure S7: Prediction results for applications of CLOUD with $L = 2$ hidden layers to data simulated under scenarios in which one tissue is evolving under Tissue Mechanism A and five tissues are evolving under Tissue Mechanism B. Each sub-panel represents a distinct pair of retention mechanisms across tissues, and all pairwise combinations of distinct retention mechanisms are considered. Violin plots display distributions of mean parameter prediction errors across the $m = 6$ tissues for each simulated test set.

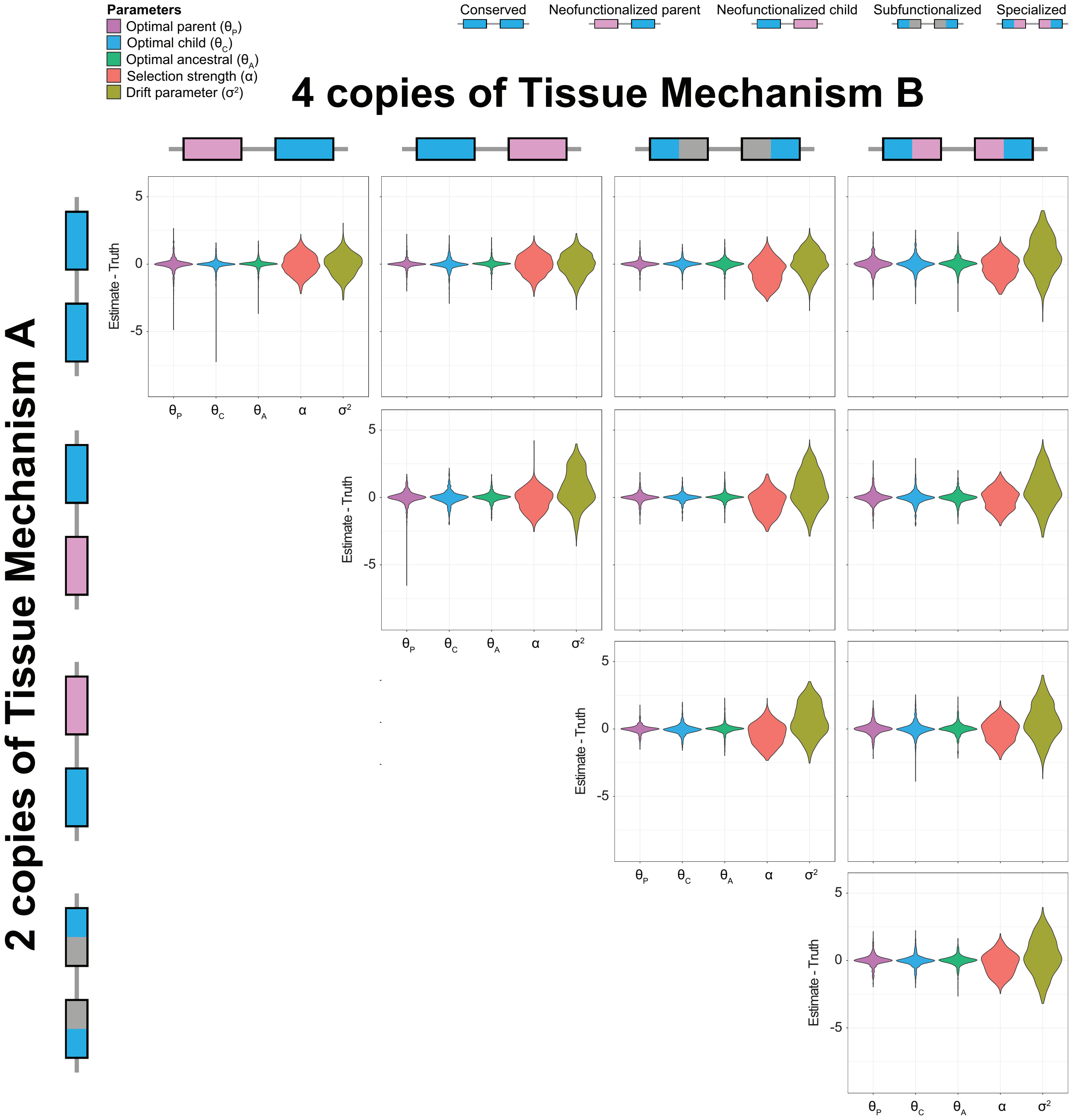

Figure S8: Prediction results for applications of `CLOUD` with $L = 2$ hidden layers to data simulated under scenarios in which two tissues are evolving under Tissue Mechanism A and four tissues are evolving under Tissue Mechanism B. Each sub-panel represents a distinct pair of retention mechanisms across tissues, and all pairwise combinations of distinct retention mechanisms are considered. Violin plots display distributions of mean parameter prediction errors across the $m = 6$ tissues for each simulated test set.

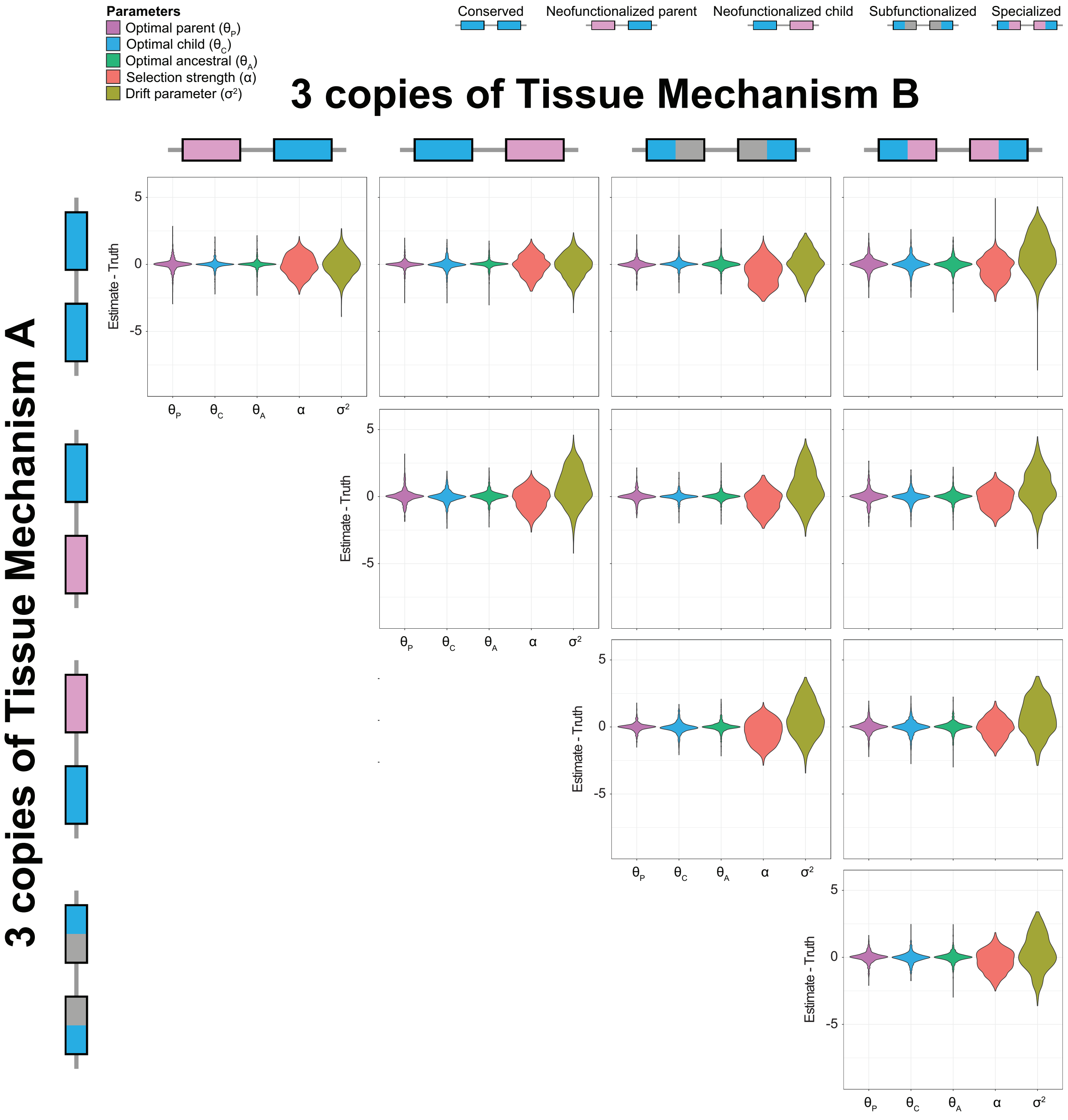

Figure S9: Prediction results for applications of CLOUD with $L = 2$ hidden layers to data simulated under scenarios in which three tissues are evolving under Tissue Mechanism A and three tissues are evolving under Tissue Mechanism B. Each sub-panel represents a distinct pair of retention mechanisms across tissues, and all pairwise combinations of distinct retention mechanisms are considered. Violin plots display distributions of mean parameter prediction errors across the $m = 6$ tissues for each simulated test set.

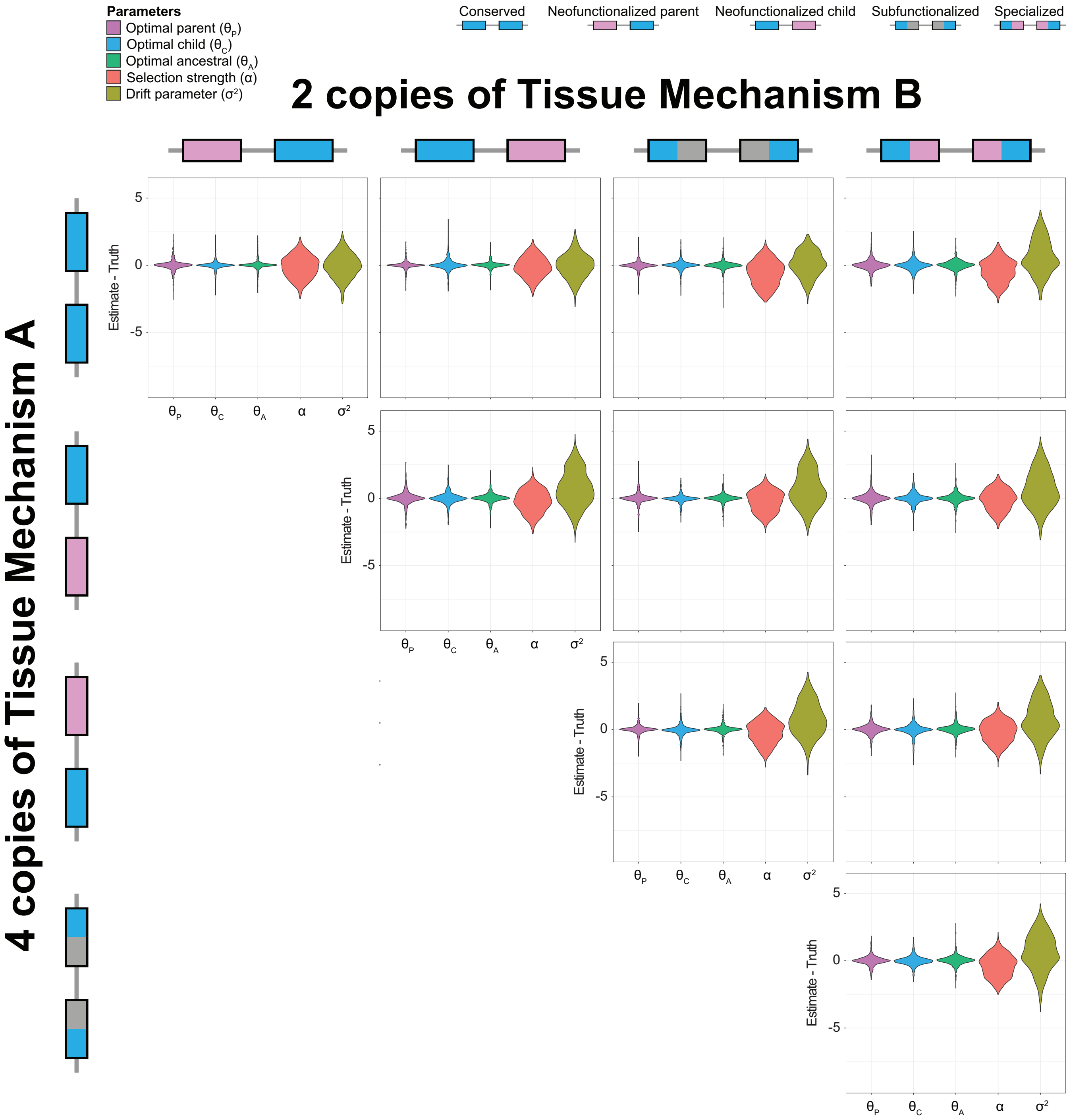

Figure S10: Prediction results for applications of CLOUD with $L = 2$ hidden layers to data simulated under scenarios in which four tissues are evolving under Tissue Mechanism A and two tissues are evolving under Tissue Mechanism B. Each sub-panel represents a distinct pair of retention mechanisms across tissues, and all pairwise combinations of distinct retention mechanisms are considered. Violin plots display distributions of mean parameter prediction errors across the $m = 6$ tissues for each simulated test set.

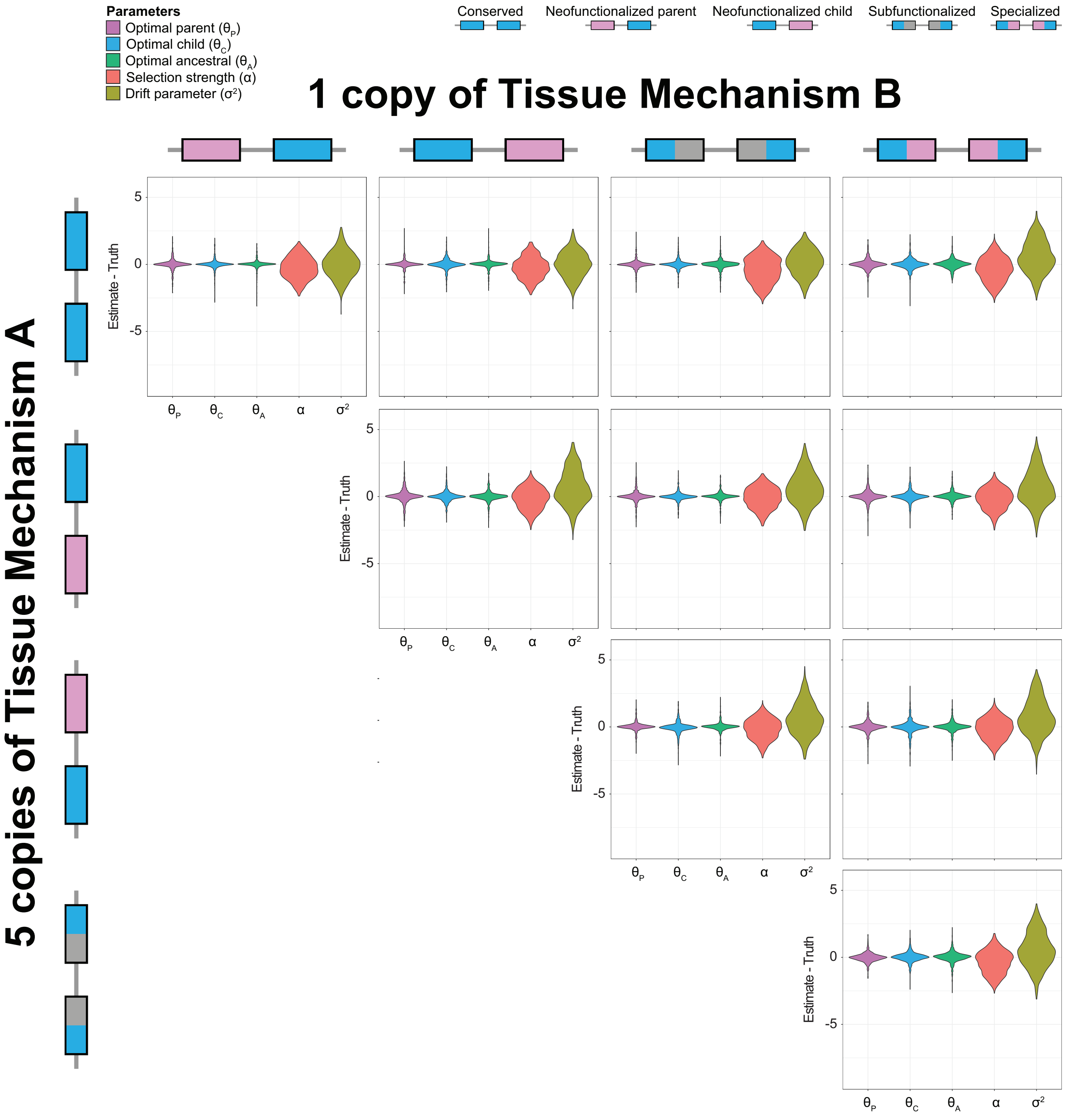

Figure S11: Prediction results for applications of CLOUD with $L = 2$ hidden layers to data simulated under scenarios in which five tissues are evolving under Tissue Mechanism A and one tissue is evolving under Tissue Mechanism B. Each sub-panel represents a distinct pair of retention mechanisms across tissues, and all pairwise combinations of distinct retention mechanisms are considered. Violin plots display distributions of mean parameter prediction errors across the $m = 6$ tissues for each simulated test set.

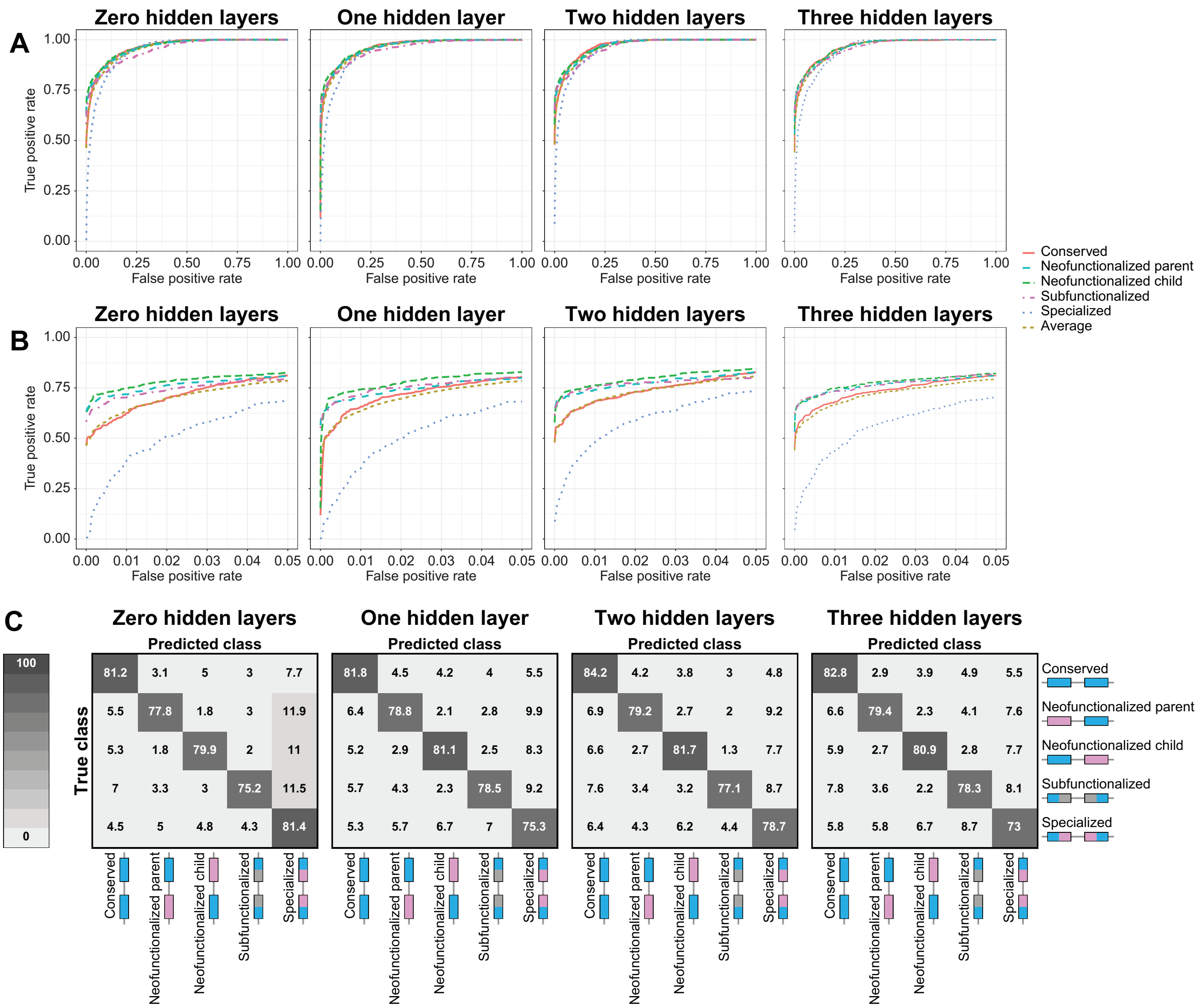

Figure S12: Classification results for applications of CLOUD with $L \in \{0, 1, 2, 3\}$ hidden layers to data simulated under parameters $\alpha \in [1, 10^3]$ and $\sigma^2 \in [10^{-2}, 10^3]$. (A) Receiver operating characteristic curves across the full range of false positive rates. (B) Receiver operating characteristic curves truncated at a false positive rate of 5%. (C) Confusion matrices depicting classification rates.

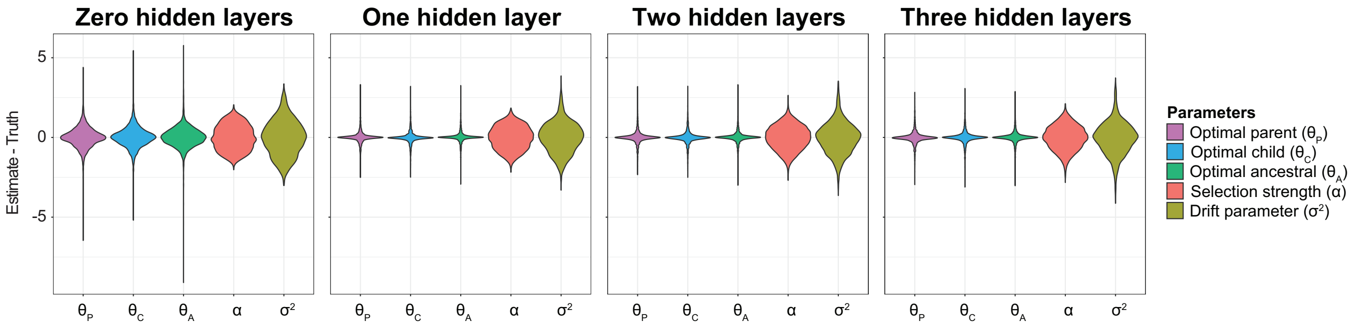

Figure S13: Prediction results for applications of CLOUD with $L \in \{0, 1, 2, 3\}$ hidden layers to data simulated under parameters $\alpha \in [1, 10^3]$ and $\sigma^2 \in [10^{-2}, 10^3]$. Violin plots display distributions of mean parameter prediction errors across the $m = 6$ tissues for each simulated test set.
